# On the interaction of fish and marine hydrokinetic turbines: insights gained through experimental and computational observations

**DOI:** 10.1098/rsos.262007

**Published:** 2026-11-11

**Authors:** Hossein Seyedzadeh, Guglielmo Sonnino Sorisio, Catherine Wilson, Mehrshad Gholami Anjiraki, Fotis Sotiropoulos, Ali Khosronejad

**Affiliations:** ^1^ Department of Civil Engineering, Stony Brook University, Stony Brook, NY, USA; ^2^ School of Engineering, Cardiff University, Cardiff, UK; ^3^ Department of Mechanical Engineering, Penn State University, State College, PA, USA

**Keywords:** fish–turbine interaction, fish behaviour, large eddy simulation, marine hydrokinetic turbine, turbulent flow, wake dynamics

## Abstract

Tidal hydrokinetic turbines offer promising opportunities for energy generation, but turbine-induced turbulence may alter fish movement, passage and habitat use. We develop an eco-hydrodynamic integration framework that maps experimentally measured fish trajectories onto turbulence-resolved large-eddy simulation fields around a vertical axis turbine. Fish behaviour is not prescribed through a behavioural model; instead, measured occupancy patterns and swimming manoeuvres are evaluated in relation to the flow field, integral length scale and estimated swimming thrust. The simulations resolve wake asymmetry, vortex shedding, shear layers and turbulence under mild and high flow conditions. Fish predominantly occupied low-turbulence regions and avoided the energetic wake and its shear layers. In some regions, fish selected relatively higher mean velocities with lower turbulence, revealing a velocity–turbulence trade-off in habitat selection. Evasive manoeuvres were frequently observed where the turbulence integral length scale approached or exceeded fish body length, while strong wake shear layers could act as hydraulic barriers to upstream passage. The results show that turbulence may reduce rotor entry while simultaneously increasing swimming effort and disrupting passage. From an engineering perspective, the framework provides a way to evaluate turbine siting, clearance, layout and operating conditions using behaviourally relevant turbulence metrics in addition to conventional performance and blade-strike criteria.

## 1. Introduction

The increasing impacts of climate change pose significant threats to ecosystems, economies and communities, underscoring the urgent need for effective mitigation strategies. Concurrently, global energy demand continues to rise annually [1], necessitating a shift towards renewable energy sources to reduce greenhouse gas emissions. Among renewables, hydropower energy technologies, including tidal and riverine hydrokinetic turbines, are distinguished for their reliability [2], offering more consistent energy system stability compared to highly variable sources such as wind and solar.

Marine hydrokinetic (MHK) turbines offer significant potential as a renewable energy source; though further technological advancements are required to match the effectiveness of wind turbines for large-scale deployment. Within hydrokinetic systems, both horizontal axis turbines (HATs) and vertical axis turbines (VATs) present unique advantages for energy capture and power generation [3]. HATs, which are widely used in experimental, numerical and pilot-scale hydrokinetic studies, are known for high power output at elevated tip speed ratios [4–7]. However, VATs offer greater operational flexibility in variable flow conditions, independence from flow direction, easy installation and reduced environmental impact on marine life [8]. Notably, VAT arrays have demonstrated greater power generation potential than HATs in twin- and multi-turbine configurations [6,9]. Müller *et al.* [8] investigated VAT wake dynamics in twin-turbine set-ups using acoustic Doppler velocimetry to measure mean velocity and turbulence characteristics. The study revealed that the presence of a second turbine significantly alters the wake structure, with counter-rotating forward configurations promoting faster wake recovery and higher downstream velocities. These results suggest that strategic turbine pairing—particularly with coordinated rotational directions—can enhance array performance by reducing wake interference. This advantage makes VATs particularly appealing for MHK applications, where minimizing environmental impact and ensuring ecological compatibility are essential considerations [9,10].

A key consideration in the deployment of MHK turbines is their potential impact on fish behaviour, particularly owing to turbine-induced turbulence. Of specific concern are the elevated levels of velocity shear, turbulent shear stresses, swirl, vorticity and high-frequency turbulent fluctuations. These features can destabilize and disorient fish, cause stress, increase energy expenditure and alter migration routes [11–13]. Turbine turbulent wakes can also influence feeding and habitat selection [14,15]. Traditional fish injury assessment (FIA) [16–18] has primarily focused on the physical interactions between fish and turbine components, with an emphasis on hydraulic stressors such as shear forces, pressure changes and blade strikes which offer important insights into direct mechanical injuries [19,20]. However FIA neglects to include the behavioural responses of fish navigating complex turbulent flows, which include avoidance behaviour and altered swimming patterns [12,21].

Eco-hydrodynamics examines the interactions between fluid flow and aquatic ecosystems, focusing on how hydrodynamic processes influence organism behaviour and habitat conditions. In MHK energy systems, turbine-induced flow features—such as turbulence and wake structures—can affect fish movement and trajectories. Incorporating this perspective allows for a more comprehensive evaluation of turbine performance alongside its ecological impacts, supporting the development of environmentally sustainable designs [22].

To date, the majority of experimental studies quantifying fish–turbulence interactions have focused on the impact of turbulence generated in the wake of a stationary obstacle. For losses in swimming stability in response to perturbations, the relationship between turbulence eddy diameter and fish length has been identified as a pertinent parameter [12,23,24]. The orientation of the obstacle and axis of rotation of their wake eddies are important; spanwise rollers (XZ plane) influence swimming stability more adversely than vertical rollers (XY plane) [12]. In addition, higher rates of destabilization occur in shear layer zones with higher magnitudes of vertical Reynolds shear stress [11].

Behavioural observations in field and laboratory settings have shown that fish tend to actively avoid turbulent wakes generated by VATs while maintaining downstream passage. Juvenile rainbow trout were observed to avoid VAT-induced wakes without experiencing delay, indicating an ability to detect and respond to turbine-generated turbulence while preserving migration pathways [25]. Similar avoidance behaviour has been documented in tidal-channel deployments, where fish preferentially avoided the rotor region, with avoidance behaviour being more prominent with higher ambient flow velocities [26]. These findings collectively suggest that turbine-induced turbulence, rather than physical obstruction, plays a dominant role in shaping fish movement near MHK installations [27,28]. Quantifying the spatial structure and energetic content of these turbulent features is therefore essential for understanding fish behavioural responses and informing the design of effective fish guidance and mitigation strategies [25,26].

Previous computational studies have employed turbulence-resolving approaches, including large-eddy simulation (LES), Reynolds-averaged Navier–Stokes (RANS) and detached eddy simulation (DES), to characterize the hydrodynamic environments generated by MHK turbines and their wakes. These studies have focused on resolving coherent wake structures, turbulence intensities and wake recovery processes that are relevant to environmental interactions, but without explicitly modelling fish behaviour. For example, numerical investigations of twin H-rotor VATs have shown that turbine rotational configuration strongly influences the self-organization of coherent wake structures and downstream flow recovery [29]. LES studies resolving turbine blade geometry have demonstrated that angular rotational speed controls blade–vortex interaction mechanisms and the spatial distribution of turbulence in VAT wakes [8]. Hybrid DES-based approaches coupled with blade element momentum models have further shown skill in reproducing mean wake characteristics and large-scale coherent structures for full-scale turbine devices, including multi-turbine configurations, while maintaining computational efficiency [30]. Collectively, these studies provide detailed descriptions of turbine-induced turbulence, but they also highlight the need to connect turbulence-resolved flow fields directly with experimentally observed biological responses rather than treating wake hydrodynamics and fish behaviour as separate problems.

The three-dimensional vortical structures generated in the wake of VATs differ markedly from those encountered in natural aquatic environments, and understanding their influence on fish behaviour remains incomplete. While previous studies have provided detailed characterization of turbine-induced wake hydrodynamics, direct links between high-energy turbulent features and observed responses of real fish are very limited [31,32]. Furthermore, the limited experimental studies [10,25,33] that examine real fish behaviour have not measured the turbine-induced flow field at the temporal and spatial resolution needed to resolve unsteady turbulent features and their properties. In this study, we address this gap by developing an eco-hydrodynamic framework that maps experimentally observed fish trajectories onto statistically converged LES fields and evaluates fish occupancy and manoeuvres against the spatial distribution of velocity and turbulence characteristics. The integration is spatial and statistical rather than time synchronized. Time-resolved spatial swimming paths of the fish are analysed in relation to resolved turbulence characteristics, including coherent vortical structures, turbulence kinetic energy (TKE) and spectral content across relevant scales. The aim is to identify high temporal energetic turbulence features and areas that influence fish response and swimming behaviour. Our findings demonstrate the capability of this integrated approach to resolve turbine-induced turbulent structures pertinent to environmental impact assessment, providing guidance for turbine design optimization, environmental impact evaluation and the development of mitigation strategies aimed at minimizing ecological disturbance in MHK applications.

This paper is organized as follows. Section 2 describes the methodology used in this paper, including the experimental details in §2.1, computational details in §2.2 and how to couple experiments and LES in §2.3. In §3, we present the results and discuss the findings of the study. Lastly, we conclude the results of this investigation in §4.

## 2. Methodology

Details of the fish–turbine interaction experiments, the computational modelling framework used to model the turbulent flow field of the fish–turbine experiments, and approaches used to understand fish response to the hydrodynamic and turbulence properties are provided in this section.

### 2.1. Experimental details

Fish–turbine experiments were conducted in a 10 m long, 0.3 m wide and deep recirculating flume located in the Hydro-environmental Research Centre, at Cardiff University, UK, with a bed slope set at 1/1000 to represent riverine bathymetry. A three-bladed, laboratory-scale VAT with diameter and height $D=H_{T}=0.12$ m was installed along the centreline of the channel, approximately 4.4 m downstream of the inlet. Experiments were conducted under two discharge conditions (mild and high flow) and two turbine operation states (stationary and rotating). The flow depth was maintained at $h_{0}=0.23$ m, with bulk velocity of $U_{\infty }=0.19$ m s${}^{-1}$ in the mild flow case and $U_{\infty }=0.25$ m s${}^{-1}$ in the high flow case featured in this paper. Juvenile rainbow trout (*Oncorhynchus mykiss*) were tested individually, with a total of $N_{\text{tested}}=20$ fish per treatment. Accordingly, the biological observations reported here are specific to this species and life stage under controlled freshwater-flume conditions.

Fish location and swimming path were recorded using a top-view PIV camera system (Baumer) at 80 fps. The planar swimming paths were then extracted using semi-automatic motion-tracking techniques. From these trajectories, fish spatial position, distance travelled, swimming velocity and spatial occupancy relative to the turbine and its wake were quantified. This paper uses the datasets relating to the time series of fish spatial position and spatial occupancy [34]. Further details of the experimental set-up, flow conditions, turbine operation and behavioural metrics are provided in Müller *et al.* [25] and Sonnino Sorisio *et al.* [10].

### 2.2. Computational details

The open channel flume and turbine experimental set-up, which is described in §2.1 was modelled using LES. The computational domain, illustrated in figure 1a, has a length of L=2.3 m in the streamwise direction. The flow depth ($h_{0}$) is set to 0.23 m in the vertical direction, corresponding to the configuration referred to as the ‘narrow flume’ [25]. The channel depth H is 0.3 m, equal to the width (B/H=1), resulting in a cross-sectional area of $A_{s}=B\times H=0.09\;{\text{m}}^{2}$.

**Figure 1.**
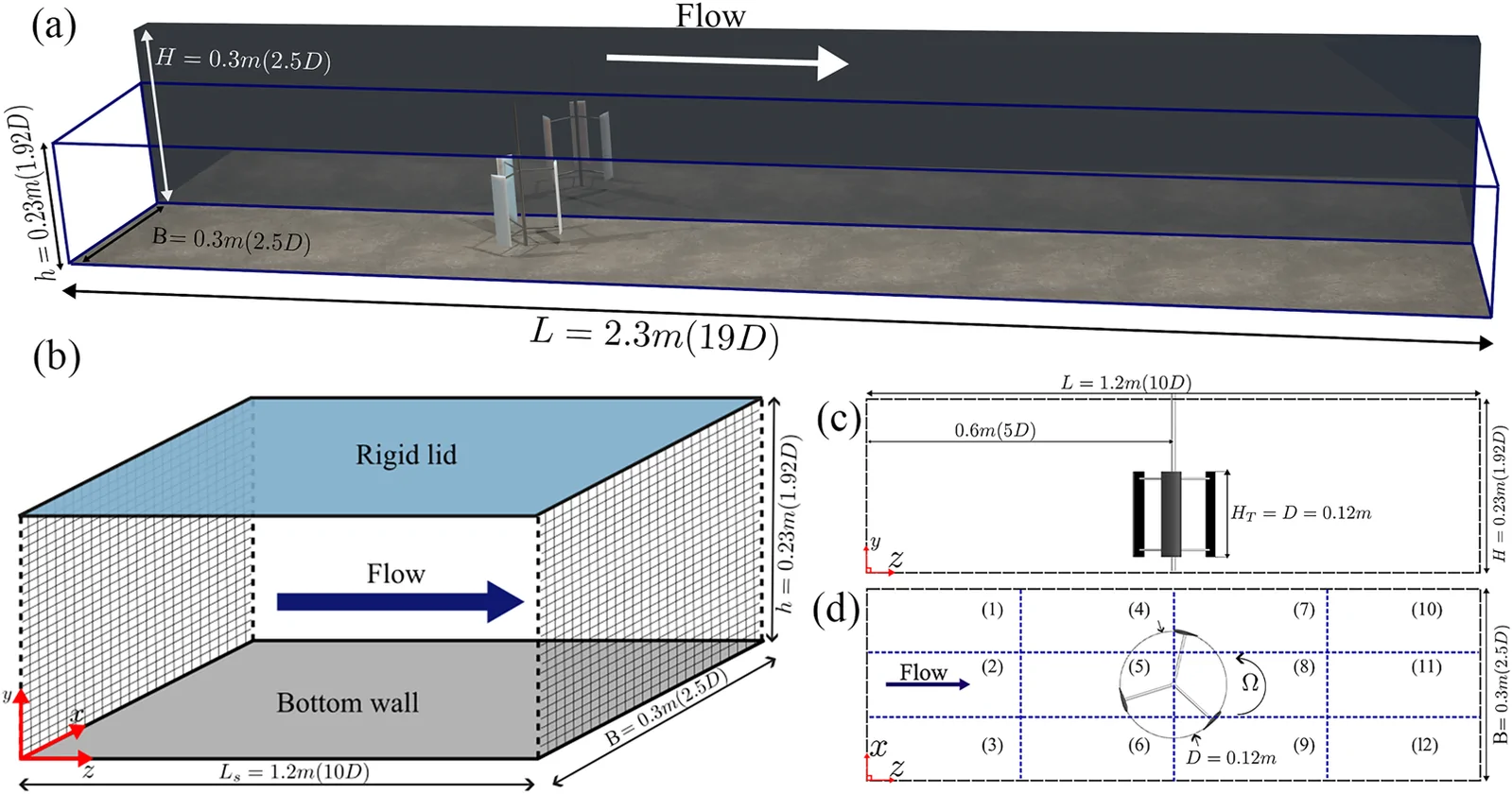
Schematic of the flume and VAT design based on the experimental set-up. (a) Three-dimensional view of the entire domain; (b) region analysed during post-processing, where fish movement is constrained using flow straighteners located five turbine diameters upstream and downstream of the turbine, covering a 1.2 m area; (c) two-dimensional schematic showing the side view; (d) top view of the domain. The domain is divided into 12 zones for further analysis.

The movement of fish in the experiment was restricted to a region bounded by two flow straighteners, as shown in figure 1b, covering a total length of 10D. To maintain consistency with experimental observations, visualizations of computational results are confined within this region. Furthermore, figure 1d provides a top-view schematic of the domain subdivided into 12 distinct subregions. These zones are used for detailed analysis of turbulence characteristics and fish behaviour in §3.

#### 2.2.1. The hydrodynamic model

The spatially filtered Navier–Stokes equations for incompressible flow, formulated in non-orthogonal generalized curvilinear coordinates, are employed to simulate the flow field [5,35,36].

LES is used as the turbulence closure model, and for the subgrid-scale stresses, $\tau_{lj}$, the dynamic Smagorinsky model is employed [37,38].

TKE (k) and Reynolds stress ($\tau_{ij}$) are defined as [39]

(2.1)k=12ui′ui′―(2.2)andτij=−ρ ui′uj′―,

where $u_{i}^{\prime }$ represents the fluctuating velocity components in the three Cartesian directions and ρ is the fluid density.

Importantly, the curvilinear immersed boundary (CURVIB) method [40] is employed to model fluid–structure interactions involving both stationary and moving objects of the flume and turbine, respectively. This method has been extended to perform RANS and LES of turbulent flows in complex environments [41,42]. In the context of the CURVIB method, the solid objects are discretized using an unstructured mesh that is embedded within the structured background grid of the fluid domain [43]. The CURVIB method treats solid boundaries as sharp interfaces, reconstructing boundary conditions at curvilinear grid nodes located near the immersed boundary by interpolating along the local boundary-normal direction.

In MHK turbine simulations, resolving the viscous sublayer using wall-resolved LES would require substantial computational resources because of the high Reynolds number. Therefore, the tangential velocity at the first off-wall point is reconstructed using the Cabot wall model [44], in which the local friction velocity is obtained iteratively from an eddy-viscosity relation with van Driest-type damping. The flow domain is discretized into 217×121×837 computational nodes in the spanwise, vertical and streamwise directions, respectively, corresponding to approximately 22 million nodes. A stretched grid provides a characteristic spacing of 1mm, or 0.0083D, around the turbine, with a representative value of $y^{+}\approx 14$ near the blades. This resolution corresponds to wall-modelled rather than wall-resolved LES. The analysis therefore focuses on outer-flow and wake-scale quantities, including mean wake deficit and recovery, wake asymmetry, major shear layers, coherent vortical structures and energy-containing turbulent motions. Near-wall quantities, including instantaneous wall shear, viscous velocity gradients, peak blade-adjacent turbulence and the precise locations of separation and reattachment, remain under-resolved and are not interpreted quantitatively. The resolution is appropriate for the eco-hydrodynamic objectives because the analysis relates fish trajectories and spatial occupancy to wake-scale velocity and turbulence distributions. The juvenile trout are approximately 60mm long, while the computed integral turbulence scales range from $0.96L_{\mathrm{fish}}$ to $4.99L_{\mathrm{fish}}$ (table 6). A grid sensitivity analysis was conducted and is presented in appendix A, demonstrating that the key wake characteristics and turbulence statistics are insensitive to further near-wall refinement. The smallest vertical grid spacing, expressed in inner wall units, is 14 (table 1). And, a non-dimensional time step ($\Delta t_{s}=\Delta tU_{\infty }/D$, where Δt is the dimensional time step) of 0.0005 was used to ensure that the Courant–Friedrichs–Lewy number remains below one.

**Table 1.** Computational grid configuration and time-step parameters used in the flow solver. Grid sizes are defined in streamwise ($N_{z}$), spanwise ($N_{x}$) and vertical ($N_{y}$) directions. Spatial resolutions are normalized by rotor diameter (D).

variable	value
$N_{x}$, $N_{y}$, $N_{z}$	217×121×837
total computational nodes	$22\times 10^{6}$
$y^{+}$	≈14
Δx/D, Δy/D, Δz/D	0.0083
$\Delta t_{s}$	0.0005
$U_{\infty }$ (m s^–1^)	0.19, 0.25
$h_{0}$ (m)	0.23
ρ (kg m^–^${}^{3}$)	998

**Table 2.** Turbine and channel specifications. The turbine diameter is denoted by D, and the blade chord length by c. The turbine solidity is defined as $\sigma =N_{b}c/\pi D$, where $N_{b}$ represents the number of blades. Ω is the angular rotational speed of the turbine. Channel dimensions are L (length), W (width) and H (depth).

MHK turbine parameters	
D (m)	0.12
c (m)	0.03
σ	0.24
$\Omega \;({\text{rad s}}^{-1})$	6.0, 8.0
$H_{T}$ (m)	0.12
hydrofoil type	NACA0015

channel description	
L (m)	2.3
W (m)	0.3
H (m)	0.3

**Table 3.** Overview of test cases detailing the flow and turbine rotation conditions. Tip-speed ratio λ and Reynolds number ReD (based on turbine diameter D=0.12m) are listed for each case. CCW denotes counterclockwise rotation.

test case	description	rotation	ReD
MS	mild flow rate, stationary	λ=0	22 800
MR	mild flow rate, rotating	λ=1.9, CCW	22 800
HS	high flow rate, stationary	λ=0	30 000
HR	high flow rate, rotating	λ=1.9, CCW	30 000

**Table 4.** Mean values of hydrodynamic and turbulent metrics computed from LES for the (a) mild-flow rotating (MR) and (b) mild-flow stationary (MS) cases.

(a)							
MR	TKE ($10^{5}$)	U ($10^{2}$)	V ($10^{3}$)	W ($10^{3}$)	$u^{\prime }v^{\prime }$ ($10^{6}$)	$u^{\prime }w^{\prime }$ ($10^{6}$)	$v^{\prime }w^{\prime }$ ($10^{6}$)
yellow	13.0	−1.4	−0.6	186.2	2.6	−3.3	−36.1
green	15.8	0.6	−0.5	187.3	−6.9	9.4	−54.8
blue	137.8	21.5	−14.9	139.1	−82.2	483.9	−118.7
purple	6.6	1.9	2.3	63.5	7.7	9.3	−2.9
red	131.0	−11.8	2.3	170.2	−112.8	195.2	−15.2
pink	131.5	21.4	0.4	105.9	−82.7	−45.9	−74.7
gold	126.5	27.1	−3.6	68.3	−159.1	−6.7	−82.2

(b)							
MS	TKE ($10^{5}$)	U ($10^{2}$)	V ($10^{3}$)	W ($10^{3}$)	$u^{\prime }v^{\prime }$ ($10^{6}$)	$u^{\prime }w^{\prime }$ ($10^{6}$)	$v^{\prime }w^{\prime }$ ($10^{6}$)
yellow	12.9	−0.5	−0.8	187.5	2.0	−2.0	−36.4
blue	16.5	5.0	−10.7	168.6	−12.7	14.2	−40.9
purple	14.4	4.6	−0.2	129.2	9.7	19.1	−14.5
red	92.9	−0.5	−4.0	173.2	14.9	159.4	9.0
pink	133.0	8.6	4.8	155.9	28.6	−151.8	86.9

**Table 5.** Estimated fish swimming speeds ($V_{f}$ in cm s${}^{-1}$) and thrust forces ($T_{f}$ in dyne) across 12 subregions for each case (HR, HS, MR and MS) from experimental data [25]. Thrust forces are calculated as $T_{f}=\rho_{f}V_{f}^{2}L_{f}^{2}$, with $L_{f}\approx 6\;\text{cm}$ and $\rho_{f}\approx 1.08\;{\text{g cm}}^{-3}$.

	high flow (HR, HS)		mild flow (MR, MS)	
region	$V_{f},T_{f}$	$V_{f},T_{f}$	$V_{f},T_{f}$	$V_{f},T_{f}$
1	43.9, 114 158.54	27.3, 29 079.58	44.2, 125 887.30	56.9, 74 836.89
2	64.5, 265 407.53	45.7, 133 277.07	53.6, 146 104.75	58.4, 232 953.59
3	79.8, 90 937.54	62.6, 48 925.33	83.8, 37 691.18	62.2, 58 214.09
4	32.4, 161 988.08	22.9, 81 364.35	26.5, 132 676.83	26.9, 161 988.08
5	77.4, 158 837.75	58.5, 94 639.39	62.4, 172 299.84	61.3, 158 837.75
6	63.9, 132 157.27	49.3, 69 408.19	58.5, 190 669.46	66.6, 132 157.27
7	67.1, 247 339.92	68.7, 152 472.73	70.4, 150 352.02	56.3, 247 339.92
8	46.3, 175 171.09	48.9, 183 284.22	35.9, 123 182.01	46.1, 175 171.09
9	38.7, 195 983.07	35.5, 79 397.23	39.2, 91 103.16	31.1, 195 983.07
10	58.3, 40 725.65	42.3, 20 466.82	52.3, 28 051.51	70.0, 93 384.00
11	71.0, 83 453.12	45.2, 92 989.34	56.9, 82 481.65	48.4, 83 453.12
12	33.5, 43 508.63	27.7, 29 909.56	32.1, 42 699.40	33.1, 40 725.65

**Table 6.** LES-computed integral length scales Λ and normalized scales $\Lambda /L_{\text{fish}}$ for all cases (HR, HS, MR and MS) at each probe. Fish length $L_{\text{fish}}=6\;\mathrm{cm}$.

	HR	HS	MR	MS
probe	$\Lambda /L_{\text{fish}}$	$\Lambda /L_{\text{fish}}$	$\Lambda /L_{\text{fish}}$	$\Lambda /L_{\text{fish}}$
P1	3.31	3.97	3.85	3.78
P2	3.66	3.93	4.38	4.51
P3	1.78	3.95	2.35	4.13
P4	3.77	2.86	3.65	3.57
P5	4.41	4.99	4.63	4.65
P6	3.65	4.59	4.38	4.80
P7	1.19	0.96	1.52	1.23
P8	2.53	2.31	2.85	2.32
P9	3.43	3.38	3.36	3.30
P10	3.92	3.93	3.17	3.15
P11	2.21	1.93	2.66	2.02
P12	3.05	3.01	2.91	3.29

The turbine is positioned at 6.25D downstream of the inlet in the streamwise direction and 1.25D in the spanwise direction, which corresponds to the centre of the channel. The turbine’s rotation is considered counterclockwise (figure 1). The parameters used in the computational model are provided in tables 2–3. Four test cases, inspired by the experimental tests [25], are simulated in this study and are summarized in table 3. One question that may arise is the relevance of the stationary turbine cases. It is important to note that there are periods when the turbine system is not operational; during these times, flow past the stationary turbine still generates a wake, which can influence fish behaviour and passage. Therefore, including stationary cases provides valuable insights into the full range of environmental impacts.

Although fluctuations in the water surface near submerged structures can introduce minor variations in the wake flow, this study employs a simplified modelling approach by assuming a rigid-lid water surface. The Froude number (Fr) is calculated to be 0.13 and 0.15 for the mild and high flow conditions, respectively. Since these Froude numbers are well below the threshold for supercritical flow (Fr<1), the flow is considered subcritical, and surface deformation effects are minimal. This justifies the rigid-lid approximation, which is commonly adopted in similar studies to reduce computational costs without significantly compromising accuracy in the near-wake region [3,4].

To define the inlet boundary condition, two precursor simulations were conducted using periodic inflow–outflow boundary conditions in the streamwise direction. Separate simulations were developed for the mild and high flow conditions to ensure accurate representation of each case. The precursor domain had cross-sectional dimensions identical to those of the inlet of the test channel and a length equal to twice the channel width (2W). The simulation was run until the total kinetic energy reached a steady state, indicating the development of a fully turbulent flow in a statistically steady condition. The velocity field from a cross-sectional plane of this precursor simulation was then extracted and applied as the inlet boundary condition for the flume simulation. At the outlet, Neumann boundary condition was imposed, ensuring appropriate treatment of both inlet and outlet boundaries.

Finally, the numerical simulations were performed on a Linux cluster equipped with 96 AMD Epyc processors. Each simulation required an average of 26 000 CPU hours to achieve statistically converged flow fields.

#### 2.2.2. The marine hydrokinetic turbine model

The physical experiments and computational simulations were conducted with a three-blade H-Darrieus VAT with a NACA0015 hydrofoil profile. The turbine has a diameter (D) of 0.12 m, a blade chord length (C) of 0.03 m and a blade height ($H_{T}$) of 0.12 m, resulting in a geometric solidity ($\sigma =N_{b}C/\pi D$) of 0.24, where $N_{b}$ is the number of the blades (table 2). The frontal area of the rotor was $A_{f}=D\times H_{T}=0.0144\;{\text{m}}^{2}$. As a result, the blockage ratio of the flow ($\beta =A_{f}/A_{s}$) was 16%. For more information on turbine details, see [8]. Further, we considered two different bulk velocities representing mild ($U_{\infty }=0.19$ m s^–1^) and high ($U_{\infty }=0.25$ m s^–1^) flow conditions, as indicated in experiments [25]. Thus, two sets of numerical simulations were performed. Additionally, for each flow condition, two angular rotational speed settings (Ω) are considered: 0 (stationary) and $3.8\;{\text{rad s}}^{-1}$, corresponding to tip-speed ratios (TSRs, $\lambda =\Omega D/2U_{\infty }$) of 0 (stationary) and 1.9, respectively. The case with λ=1.9 corresponds to the optimal TSR ($\lambda_{\text{opt}}$).

### 2.3. Eco-hydrodynamic integration of large-eddy simulation with experimental live-fish trajectories

The purpose of the integration framework is to connect experimentally observed fish behaviour with the spatial distribution of turbine-induced hydrodynamic features. Digitized fish positions were mapped onto the computational domain and evaluated against LES-derived velocity and turbulence metrics at corresponding locations and flow regions.

Fish trajectories recorded using the top-view camera in the experimental study [10,25] were digitized at 4 Hz using the open-source Animal Tracker plugin in ImageJ. For video 1 in the supplementary material, the fish positions and body outlines were reconstructed in Blender^1^ and resampled at intervals of 0.9917 s over the 600 s experimental record. The experimental trajectory was displayed together with instantaneous LES-computed Q-criterion isosurfaces coloured by instantaneous velocity magnitude.

The experimental and numerical datasets were generated independently and were not temporally synchronized. However, the LES reproduced the experimental geometry, flow depth, bulk velocity, turbine operating state and tip-speed ratio and was sampled after statistically converged conditions were established. The coupling is therefore spatial and statistical: time-averaged and regionally averaged LES quantities characterize the representative hydrodynamic environment for each experimental treatment, while the instantaneous LES fields in the supplementary video provide representative wake visualizations rather than the exact flow experienced by an individual fish. Details of the tracking, validation and video construction are provided in appendix B.

Statistical analyses were used to evaluate relationships between fish position and the velocity and turbulence metrics resolved by the LES. The analyses were performed in RStudio using R version 4.2.2 (http://www.R-project.org). Data distributions were first assessed for normality, after which generalized linear models (GLMs) were fitted using the lme4 package [45]. The time spent in each channel subregion was normalized by the corresponding subregion area. The GLMs included subregion, fish standard length and fish mass as explanatory terms. Gaussian GLMs provided the most suitable residual distributions for the MS and MR cases. Single terms were assessed using the drop1 function and Akaike information criterion, and Tukey-adjusted pairwise comparisons among subregions were performed using the emmeans package [46].

Fish swimming velocity was calculated separately from the x- and y-coordinates contained in the experimental dataset sampled at f=27 Hz. The displacement between consecutive positions, Δx and Δy, was divided by the corresponding time interval, Δt=1/f, according to

(2.3) $$V_{\mathrm{fish}}=\frac{\sqrt{\Delta x^{2}+\Delta y^{2}}}{\Delta t}.$$

The resulting quantity represents the absolute planar swimming velocity and is used in §3.2.3 for further analysis.

## 3. Results and discussions

The turbulent flow downstream of the turbine generates an unsteady three-dimensional flow environment characterized by shear layers, vortices and velocity gradients. Such an environment has the potential to disorient or physiologically stress fish. The fish can sense turbulence with their lateral line system—i.e. a sensory organ capable of detecting viscous hydrodynamic signals around the body [47]. Under different turbine operating conditions, the resulting turbulent flow fields transmit varying hydrodynamic signals to fish, prompting different behavioural responses. By analysing the experimental data alongside the high-fidelity LES results, we aim to understand these behavioural variations from a fluid dynamics perspective.

### 3.1. Turbulent wake dynamics

In figure 2, we present the LES results of instantaneous and time-averaged velocity magnitude contours for all test cases. As seen, the LES results reveal distinct flow characteristics in the downstream region of the VAT, including wake asymmetry and complex vortical structures. The wake asymmetry is clearly evident in this figure and it is consistent with prior observations of rotating VATs [8,25]. It is often attributed to the presence of highly turbulent vortices on the advancing side (windward) of the turbine compared to the retreating (leeward) side, owing to the dynamic stall effects [5,48–50]. As the blade advances against an adverse pressure gradient, severe flow separation occurs, leading to stronger vortex shedding. Consequently, the majority of power production takes place on the advancing side, where the momentum deficit is greater. The resulting vortices are clearly captured in our simulations, highlighting the differences between stationary and rotating conditions (figure 2).

**Figure 2.**
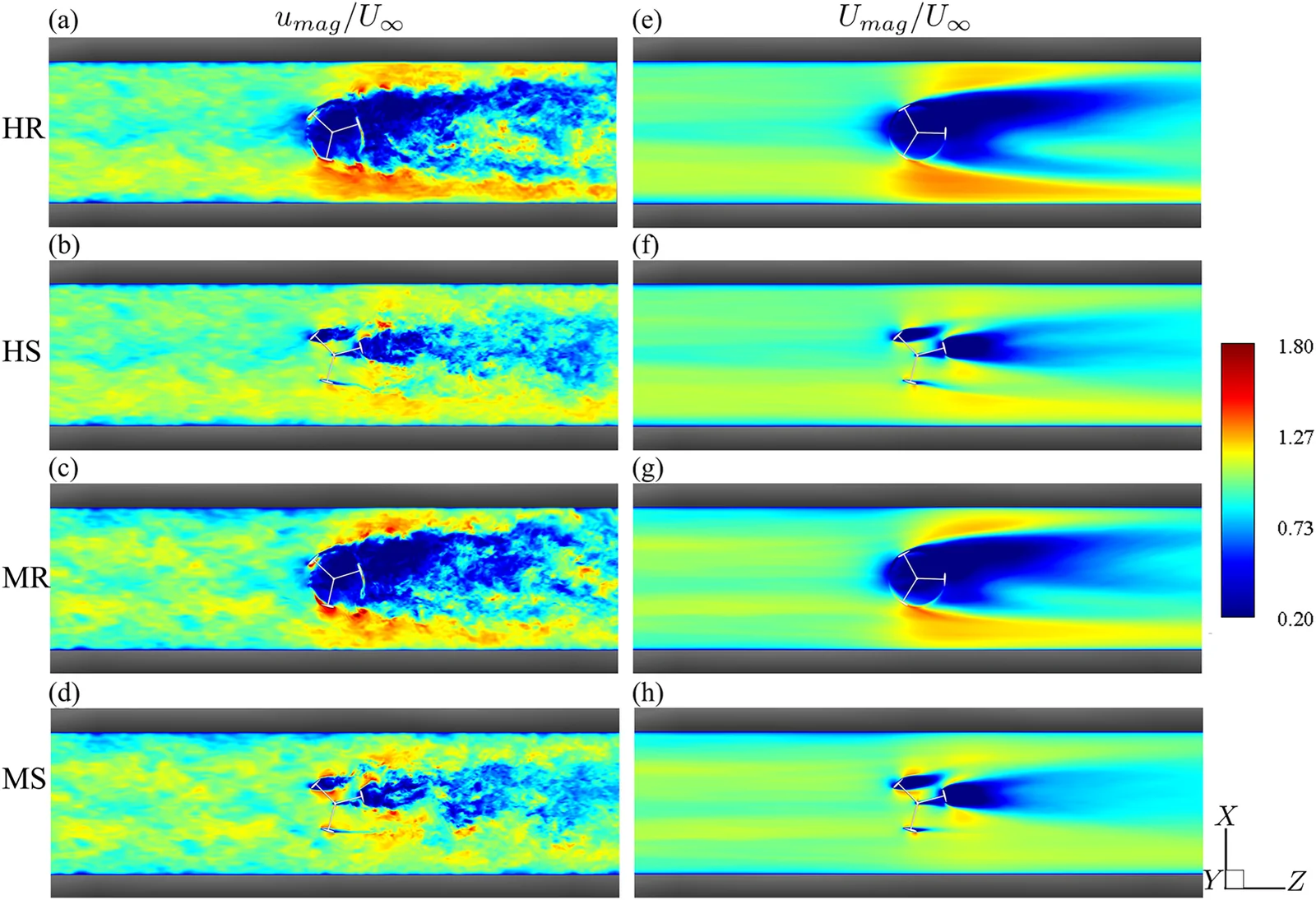
Contour of instantaneous ($u_{\text{mag}}$) and time-averaged ($U_{\text{mag}}$) velocity magnitudes, normalized by the bulk velocity ($U_{\infty }$). Contour plots are shown on horizontal planes 0.5D above the bed. (a,e) HR, (b,f) HS, (c,g) MR, and (d,h) MS. Flow direction is from left to right.

Figure 3 illustrates the vertical velocity component throughout the water column, as obtained from LES, highlighting regions of strong vertical flow and notable variations across different test cases. These vertical flow patterns further contribute to the observed wake asymmetry and add complexity to the VAT’s wake dynamics, potentially influencing fish behaviour and interactions within these regions.

**Figure 3.**
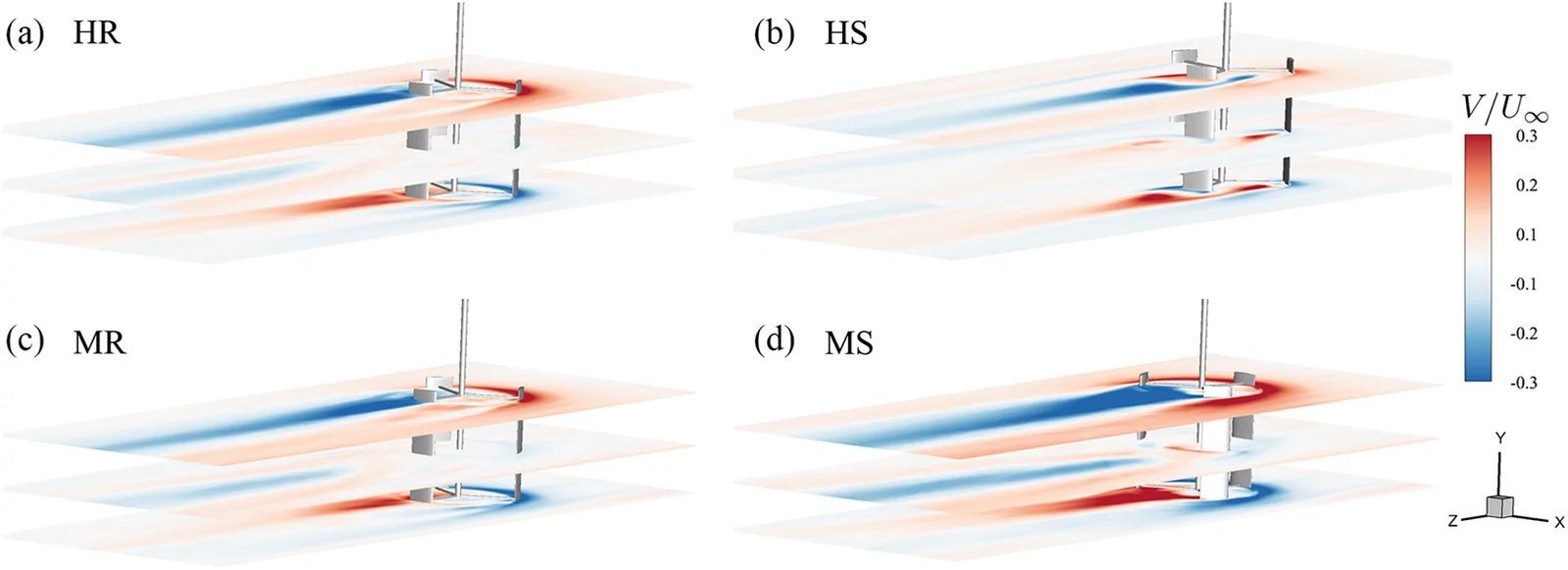
Contour maps showing the vertical component of time-averaged velocity (V), normalized by the bulk velocity ($U_{\infty }$) at different flow depths. Namely, the top, mid and lower planes are taken from y=0.92D, 0.66D and 0.25D from the bed, respectively. (a) HR, (b) HS, (c) MR, and (d) MS. Flow direction is from right to left.

The vorticity analysis from LES, shown in figures 4 and 5, highlights the periodic nature of vortex shedding, which predominantly occurs on the advancing side of the rotating VAT rotor. In particular, figure 4 presents the instantaneous and time-averaged normalized out-of-plane vorticity contours ($\omega_{y}$ and ${\overset{―}{\omega }}_{y}$, respectively) for all cases. As seen, the colour maps illustrate the periodic shedding of vortices from the blades, driven by dynamic stall effects. Further, the wake asymmetry is clearly evident in the rotating turbine and becomes more pronounced further downstream of the turbine. On the advancing side, vortices exhibit clockwise rotation (negative vorticity), while on the retreating leeward side, the vortices rotate counterclockwise (positive vorticity).

**Figure 4.**
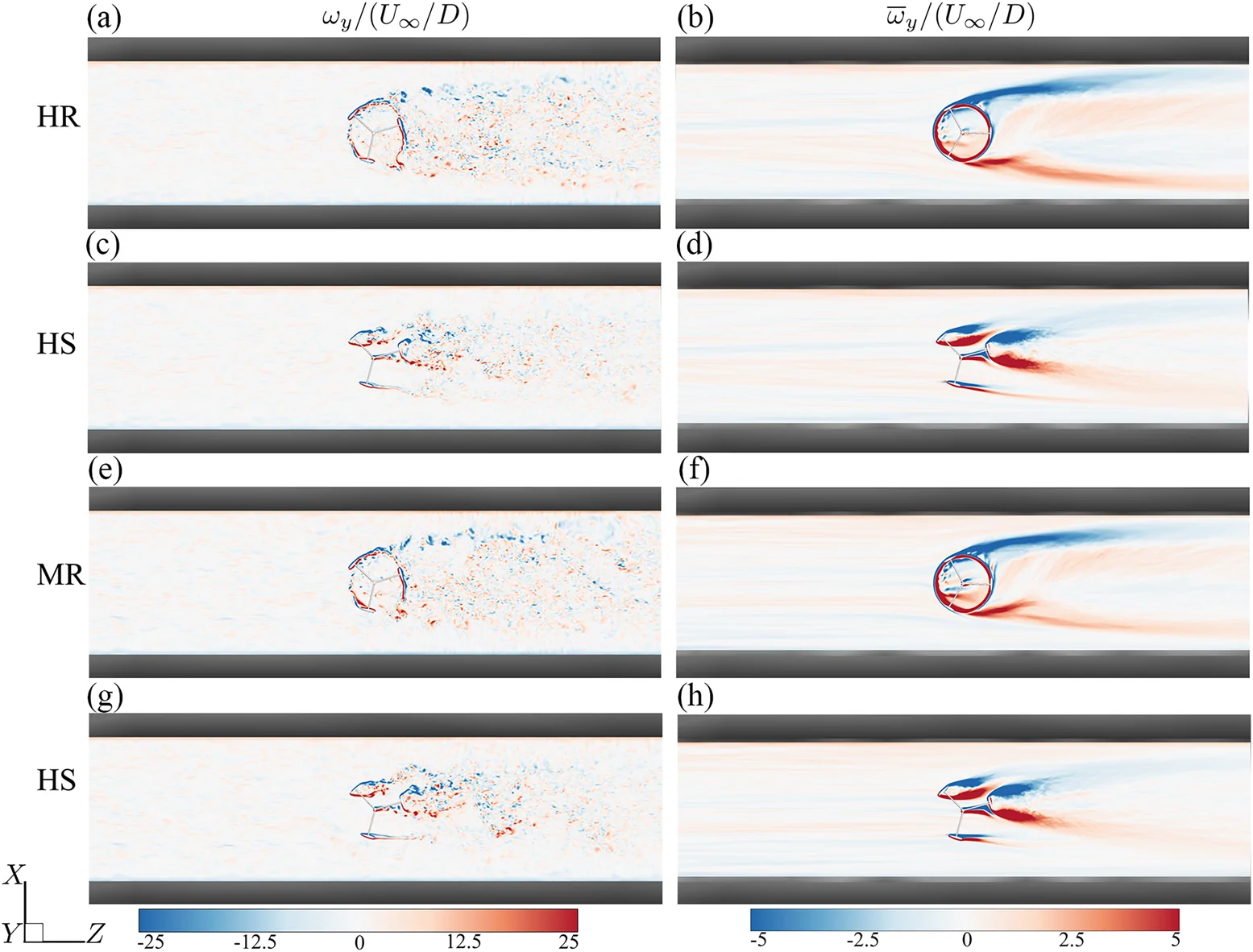
Colour maps of instantaneous and time-averaged out-of-plane vorticity ($\omega_{y}$ and ${\overset{―}{\omega }}_{y}$, respectively). Contour plots are shown on horizontal planes 0.5D above the bed. (a,b) HR, (c,d) HS, (e,f) MR, and (g,h) MS. Flow direction is from left to right.

**Figure 5.**
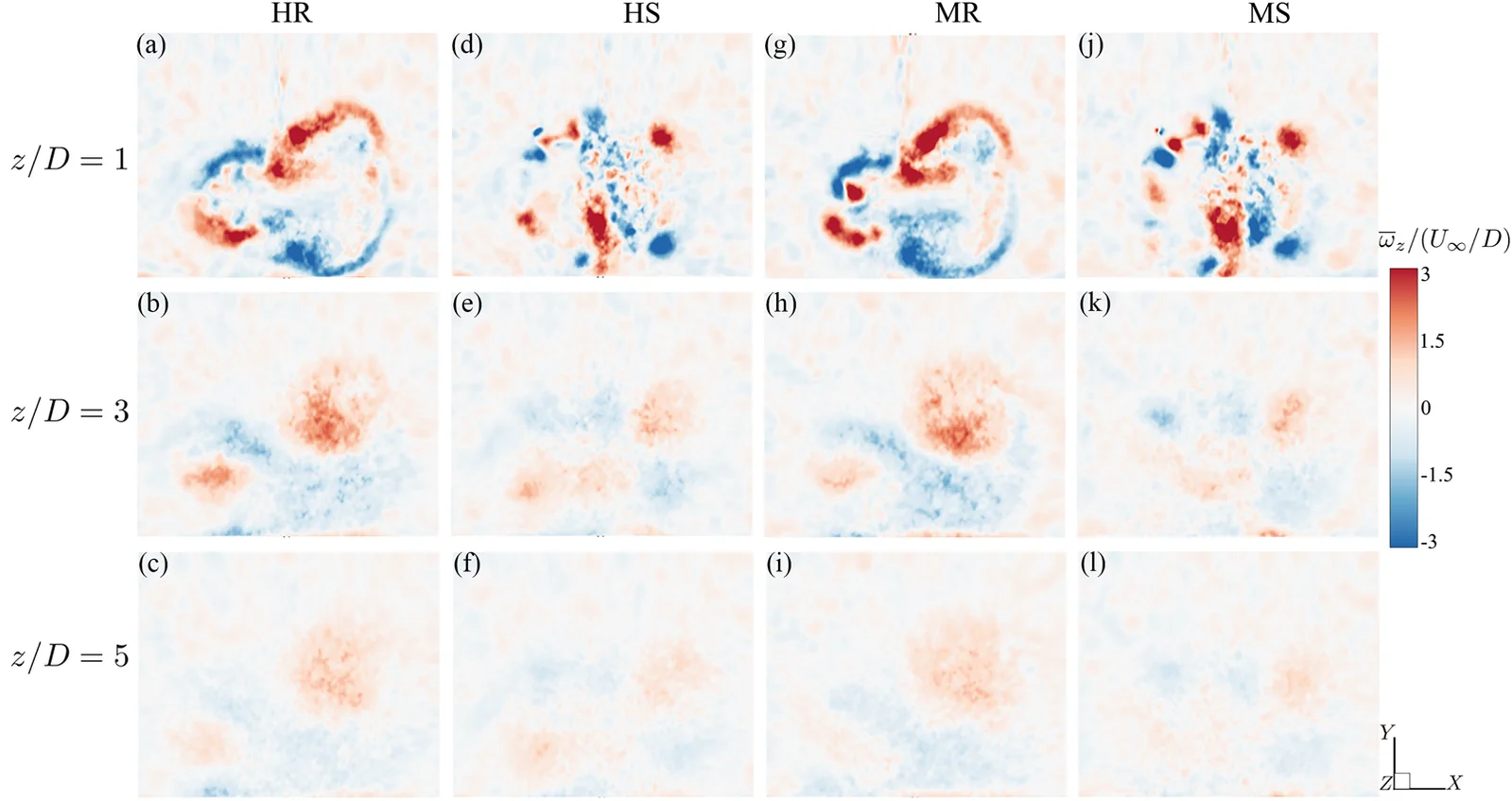
Colour maps of the normalized time-averaged out-of-plane vorticity (${\overset{―}{\omega }}_{z}$) on cross-sections perpendicular to the flow direction. (a–c) HR, (d–f) HS, (g–i) MR, and (j–l) MS.

Figure 5 displays the time-averaged out-of-plane vorticity ($\omega_{z}$) on cross-sections perpendicular to the flow direction. Counter-rotating vortices (CRVs) are especially prominent in the rotating cases, obtained from LES. These flow structures are often observed in engineering applications [51] and are a key characteristic of swirling flow patterns [52,53]. The vorticity fields are marked by a general symmetry, although interactions with the shaft and strut wakes introduce minor asymmetries. As the flow progresses downstream, the CRVs begin to merge together, while the dominant vortices persist. These vortex dynamics results in a broader wake region with reduced vorticity field, forming the core structure of the VAT-induced wake.

The vortex dynamics observed in figures 4 and 5 contribute to the formation of periodic three-dimensional vortical structures, which are visualized using the isosurfaces of the Q-criterion (= 50). These isosurfaces are overlaid on velocity magnitude contours, using distinct colour schemes to highlight regions of high-speed flow around the isosurfaces. As seen in figure 6, the isosurfaces of Q-criterion reveal highly turbulent regions surrounding the blades, shaft and struts, where distinct tip and spanwise vortices are shed. Under rotating conditions, vortex development is primarily concentrated on the advancing side of the rotor, owing to dynamic stall, with limited vortex activity observed on the leeward side. By contrast, the stationary conditions produce vortical structures mainly owing to the interaction between the incoming flow and the turbine blades. These visualizations not only reveal turbulence complexity but also offer critical insights into how such structures may influence fish behaviour, energy expenditure and ecological interactions in turbine-affected environments.

**Figure 6.**
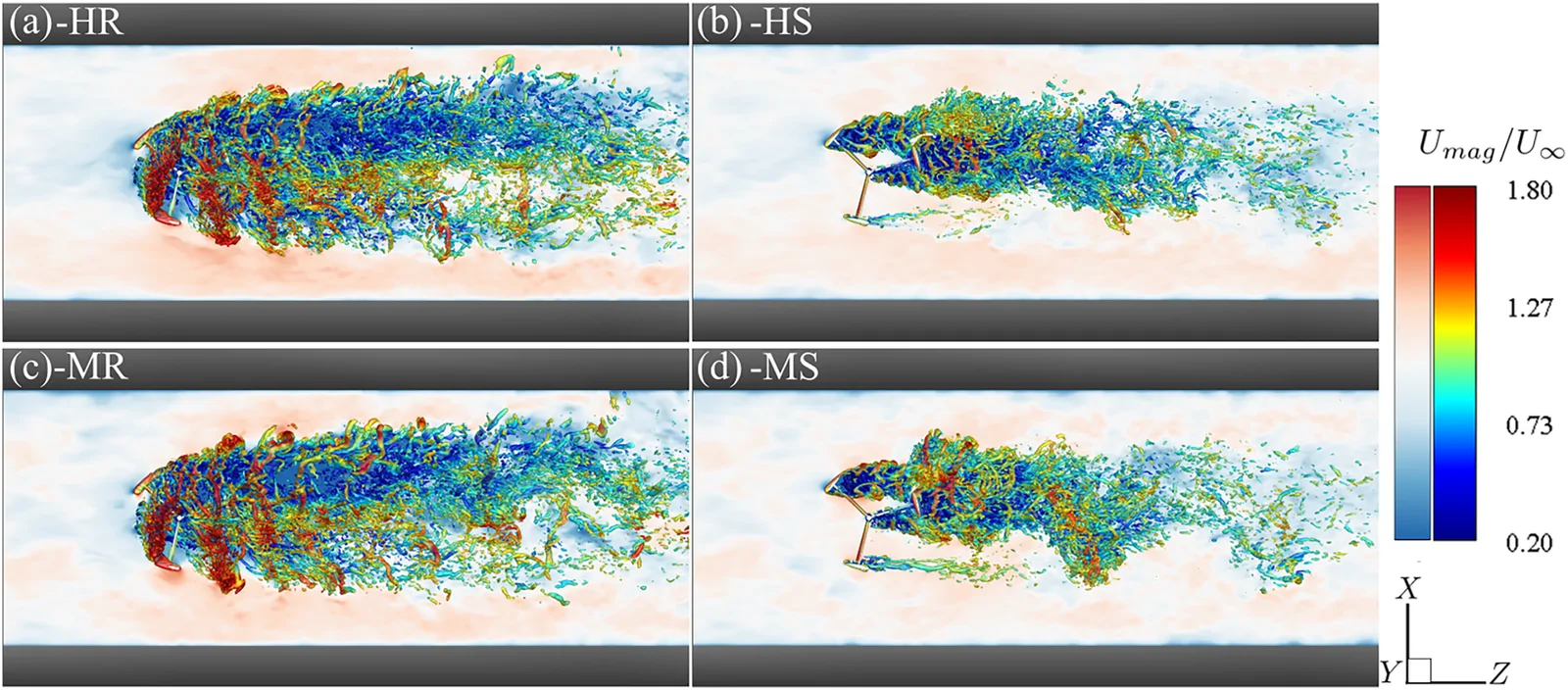
Snapshots of Q-criterion isosurfaces (Q=50), coloured by velocity magnitude, are shown for all test cases: (a) HR, (b) HS, (c) MR, and (d) MS. These three-dimensional visualizations are embedded with contour maps of instantaneous velocity magnitude ($u_{\text{mag}}$), normalized by the bulk velocity ($U_{\infty }$). Contour plots are shown on horizontal planes 0.5D above the bed. Flow direction is from left to right.

These results characterize the turbine wake independently of the biological observations and define the hydrodynamic environment used in the integration analysis. In the following section, the experimentally observed fish behaviours are integrated onto the resolved flow.

### 3.2. Eco-hydrodynamic for interpretation of experimentally observed fish behavioural responses in turbulent environments

The current FIA framework primarily considers mechanical injuries caused by direct contact with turbine blades (blade strikes) as the main source of injury to fish [19,20]. However, our integrated LES-based numerical and experimental approach examines fish behavioural responses to the turbine-induced flow field through statistical and spatial analyses. We delineate and partition *flow regions* based on the time fish spent in each region and undertake statistical analysis to correlate occupancy time with the hydrodynamic and turbulence characteristics of those regions. Fish swimming speed in the different flow regions in relation to occupancy time is also examined. Flow regions are defined at a high spatial resolution scale to examine the hydrodynamic and turbulence impact of the flow field and a lower spatial scale to understand avoidance/preference behaviour in each flow region. Five behavioural responses are considered: (i) passing, which refers to fish moving from downstream to upstream of turbine; (ii) near pass, i.e. when fish attempt to move upstream but eventually drift back downstream; (iii) entering, which describes fish entering the rotor area; (iv) evasion, characterized by sudden changes in swimming direction as fish attempt to avoid the turbine; as well as (v) strike.

#### 3.2.1. Statistical analysis of time spent in each area and linkage to hydrodynamic and turbulent metrics

An analysis of time spent by fish in areas based on velocity and turbulent metrics is presented in figure 7 and table 4. In the MR case, the fish spent more time in the purple area than all areas except for the red area (figure 7, GLM, *p* < 0.0156), indicating a preference for relatively lower magnitudes of TKE, Reynolds shear stress and flow velocity by remaining close to the channel walls and downstream corners and away from the turbine. The purple area has the lowest values of almost all hydrodynamic metrics tested in the MR treatment with the exception of U, V and $\overset{―}{u_{i}^{\prime }v_{i}^{\prime }}$ for which the yellow area has the lowest values. For the MS case, turbulence metrics for the different areas are generally lower than for MR. The fish in this test case (MS) displayed a preference for the purple area over the pink (stationary turbine wake, figure 7, GLM *p* = 0.047) but no other significant difference in preference existed between other areas. In the MS case, the pink area had significantly higher values of all hydrodynamic and turbulent criteria than the purple.

**Figure 7.**
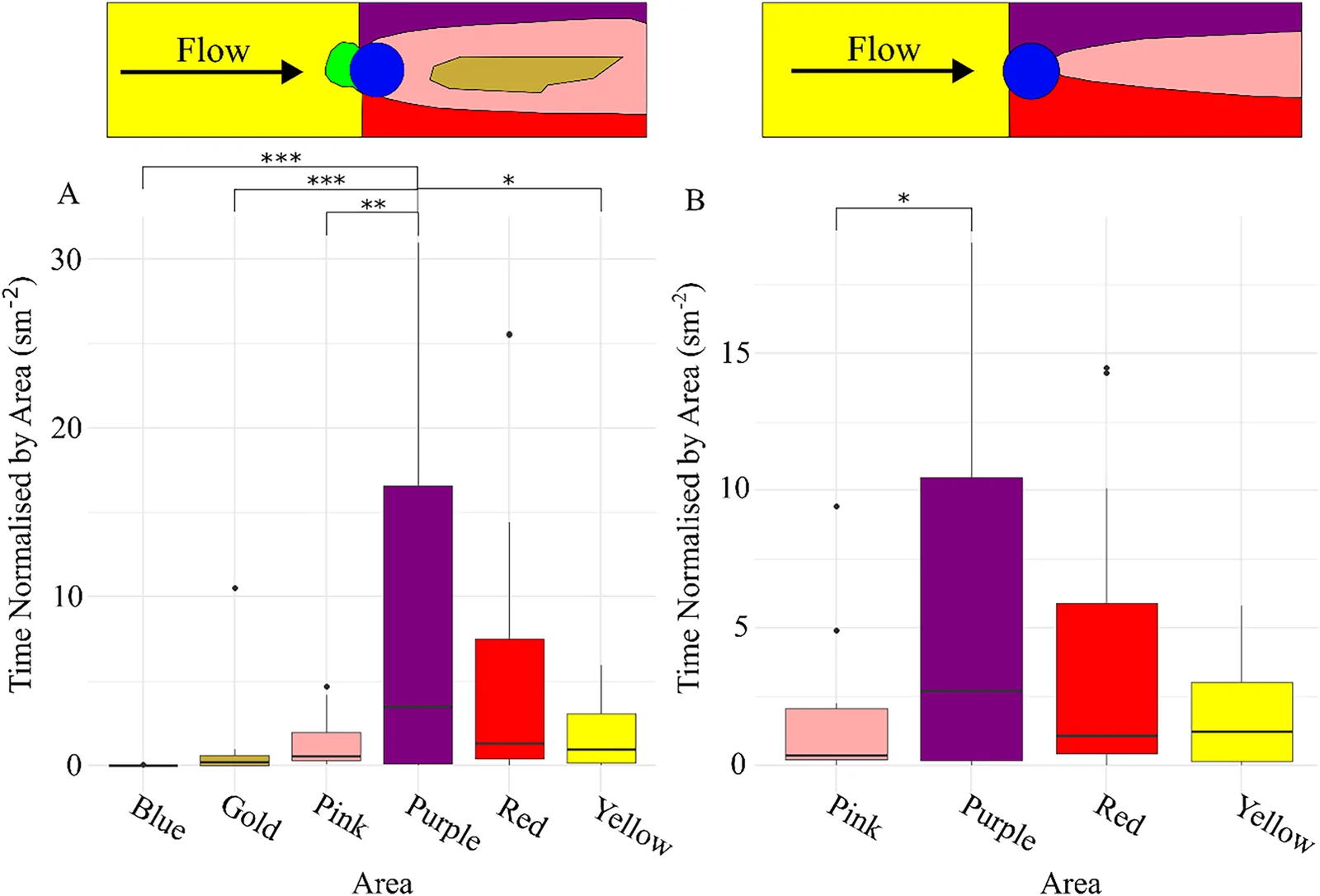
Boxplots showing time spent in each area normalized by area for each case. Above each plot is a diagram showing how areas are partitioned for each case: (A) results for the MR case; (B) results for the MS case. For all boxplots, the mean, interquartile range are represented with the whiskers showing the 95th percentile. ^∗^p≤0.05, ^∗∗^p≤0.01 and ^∗∗∗^p≤0.001 (p<0.05 is considered statistically significant). Table 4 contains the mean values of the considered hydrodynamic and turbulent metrics.

In MR, the fish spent significantly more time in the purple area, and this can likely be attributed to the reduced turbulent metrics in this area compared to most of the other areas. The preference for the purple over the yellow area in the MR case can be explained by considering that the turbine wake impacts the upstream passage [25]. The difference between yellow and purple areas could therefore be a combination of hydrodynamic preference and a behavioural reticence to pass upstream of the turbine. The MS case showed that the fish preferred the purple area to the pink area, and this is also most likely associated with the higher turbulence in the turbine wake of the stationary turbine. The area occupied by the turbine in MS was entirely avoided by the fish despite the turbine being stationary, potentially showing some behavioural dissuasion or avoidance of the vortex shedding by the fish in this area.

#### 3.2.2. Spatial preferences and avoidance

To examine how fish behave in response to wake turbulence as a stressor, we juxtapose heat maps of the fish position with LES computed Reynolds stress $\overset{―}{u^{\prime }w^{\prime }}$ and TKE over the horizontal plane at mid-depth 0.5D in figure 8. The heat map of fish position reflects the relative amount of time spent by all fish at the precise location, expressed as a normalized density. This figure indicates that fish spent most time in the downstream corners of the channel, along the channel sidewalls and downstream wall; a small amount of time in the turbulent wake and shear layers immediately downstream of the rotating turbine (figure 8; HR and MR). Owing to the strong asymmetry of the turbine wake (figure 8; HR), preference is shown for the right-hand sidewall (looking in the downstream direction). Furthermore, fish spent some time in the bow wake of a rotating turbine (HR and MR) compared to their stationary counterparts (HS and MS).

**Figure 8.**
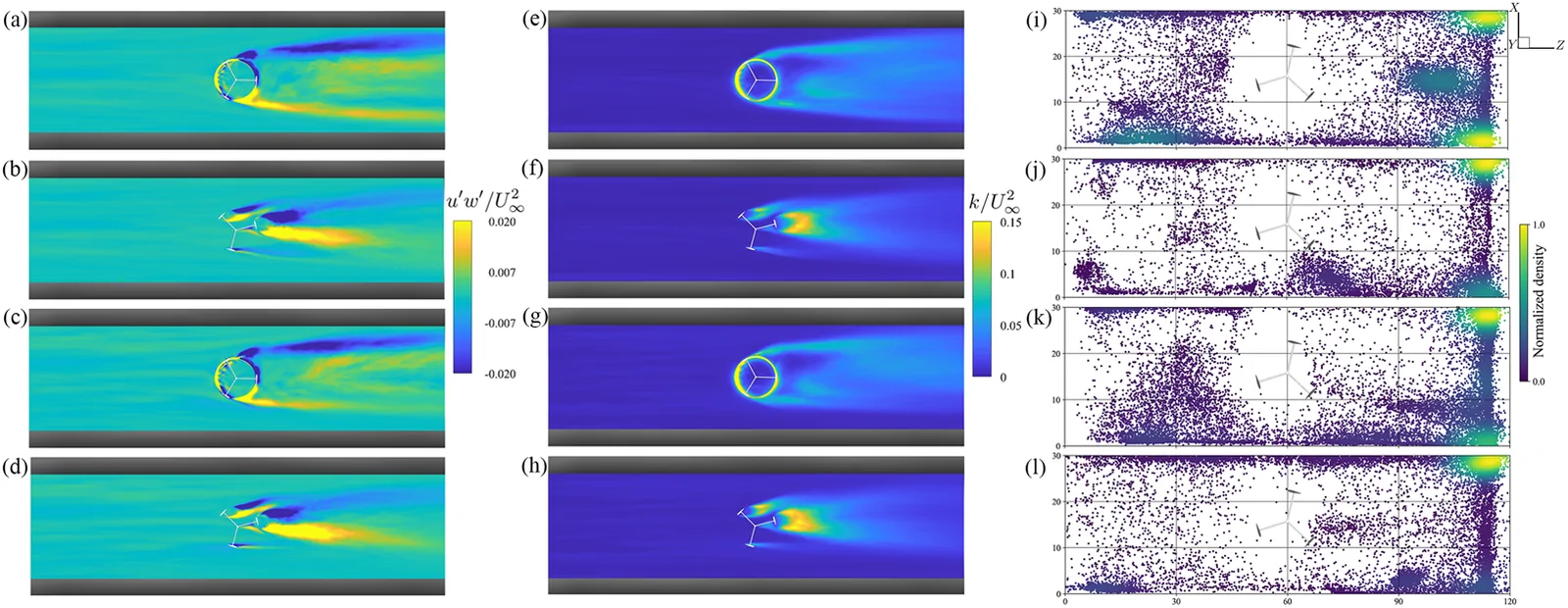
LES-computed contours of Reynolds stress ($(u^{\prime }w^{\prime })/U_{\infty }^{2}$) and TKE ($k/U_{\infty }^{2}$), together with experimentally measured fish position distributions. (a–d) The Reynolds stress contours, (e–h) the TKE (k) contours and (i–l) the corresponding scatter-density maps of fish positions. The contour plots are extracted on a horizontal plane located at 0.5D above the bed. The four rows correspond to the HR, HS, MR and MS operating conditions, respectively. In (i–l), point colour represents the normalized fish density, where brighter colours (yellow/green) indicate regions of higher fish presence and darker colours indicate lower density. Flow direction is from left to right.

To illustrate the spatial relationship between fish movement and turbine-wake structures using our integrated experimental and numerical approach, figure 9 presents the experimental trajectory of a representative fish in chronological order, superimposed onto representative instantaneous LES fields for the MR case. The LES wake is visualized using Q-criterion isosurfaces (Q=50), coloured by instantaneous velocity magnitude. The red circle in each panel marks the fish position at the preceding experimental time step. The experimental fish trajectory and LES fields were not temporally synchronized. Accordingly, the instantaneous structures shown in each panel represent characteristic wake realizations under the MR operating condition rather than the exact structures encountered by the fish. In figure 9a, the fish is released from the downstream right corner of the channel, a location frequently used for station holding behaviour. The downstream corners of the channel and areas near the sidewalls are commonly used for near-pass or passing behaviours. In figure 9g, the fish performs an overturning manoeuvre while entering the wake region, which the LES characterizes as containing energetic coherent structures. The combined snapshots are presented in video 1 in the supplementary material to illustrate the spatial relationship between the measured fish trajectory and representative turbine-wake structures.

**Figure 9.**
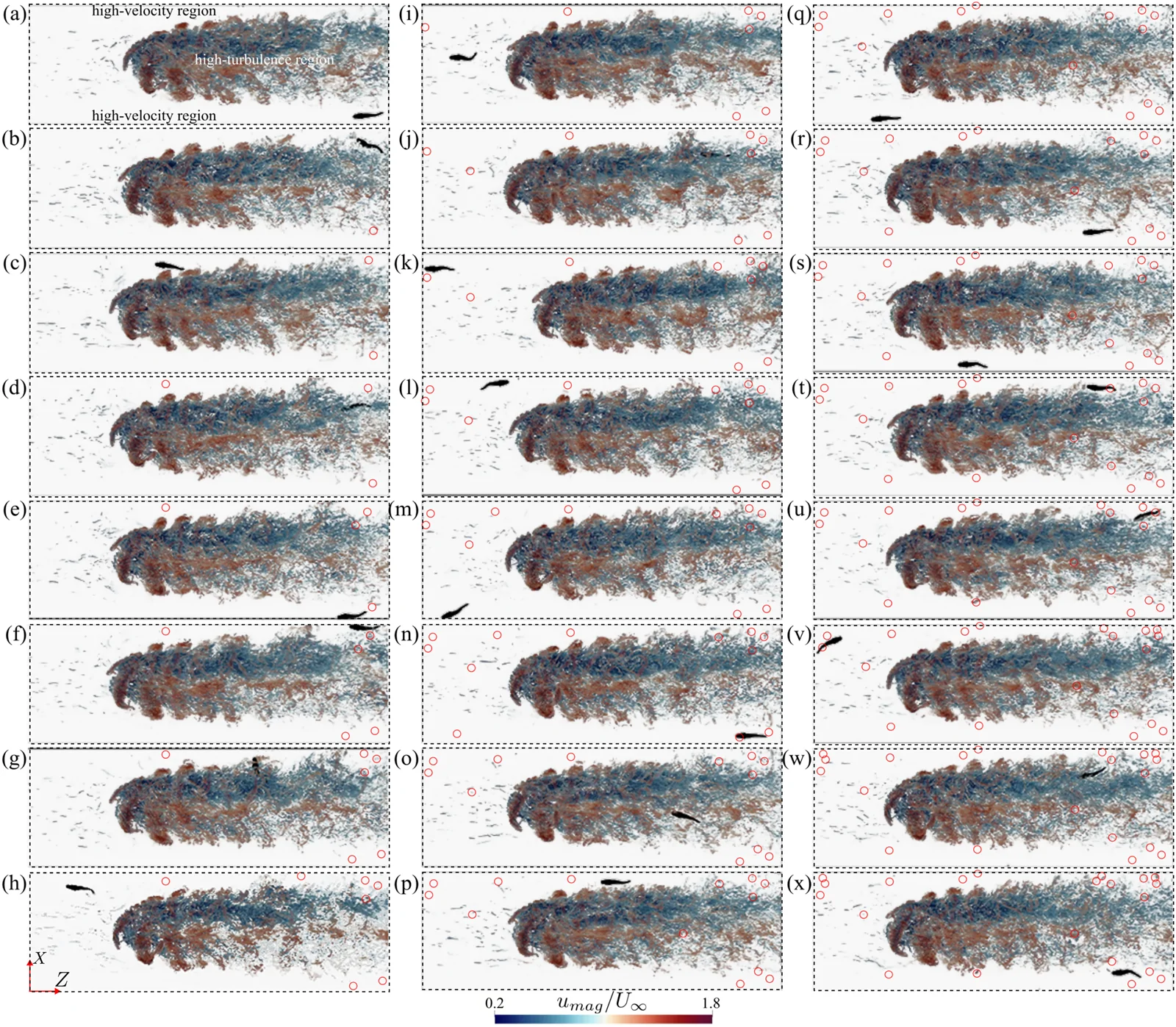
Time-resolved snapshots of fish trajectories digitized from experimental observations and superimposed on LES-computed isosurfaces of the Q-criterion, coloured by normalized velocity magnitude $\left(u_{\text{mag}}/U_{\infty }\right)$. The fish depicted in the figure is a real fish, as explained in §2.3. The sequence of panels (a–x) spans the full record length of 600 s, with each snapshot representing 0.9917 s of real time. The red open circles mark the time history of the fish position during the test, highlighting the fish positioning and its manoeuvre avoiding highly turbulent regions. Flow direction is from left to right. The fish positions follow the experimental chronology, whereas the LES fields are representative instantaneous realizations under the same operating condition and are not temporally synchronized with the experiment.

The experimental observations highlight several key behavioural trends emerging from fish movement patterns under varying turbine operation and flow conditions. For example, fish response to turbine-induced turbulence includes both avoidance behaviours and distinct swimming strategies that are closely linked to the wake dynamics discussed in §3.1. Our analysis linking both fish position density and time spent to the LES-resolved turbulence field confirms this quantitatively; fish predominantly avoid regions with high TKE and strong shear layers that delineate the boundaries of the rotor wake (figures 8 and 9). These results indicate that fish occupy subregions with lower turbulence intensity outside the rotor wake, particularly near the sidewalls and downstream corners of the channel. It can be observed that fish perform station holding in regions away from high-energetic vortical structures (figure 9a,e,n,x). It is evident that fish prefer higher mean velocity regions over areas dominated by energetic eddies (figure 6). Under typical conditions, fish tend to occupy low-velocity regions to minimize the energetic cost of station holding. However, when low-velocity zones are characterized by elevated TKE and strong vortical structures (e.g. shear layers and coherent eddies), maintaining stability becomes more energetically demanding. In such cases, fish appear to preferentially occupy regions with relatively higher mean velocity but lower TKE, where the flow is more uniform. This behaviour suggests a velocity–turbulence trade-off, in which fish select flow regions that minimize the combined energetic cost associated with both swimming effort and maintaining stability in turbulent flows.

We examined the TKE to explore its connection with regions where fish were frequently observed. As noted earlier, experimental observations show that fish tend to remain near the sidewalls and, more specifically, at the downstream flow straightener (figure 8). The LES-computed TKE reveals a marked reduction in these areas and further downstream of the turbine, forming relatively calm regions. These low-turbulence zones coincide with the observed spatial distribution of fish occupancy, while the more turbulent central channel corresponds to areas they largely avoid. The measured fish trajectories and the computed turbulence map in figure 9 further clarify this relationship between turbulence levels and fish ‘safe zones’ downstream of the turbine.

Interestingly, fish seldom occupied the central region of the wake, despite its relatively lower mean velocity, likely owing to the presence of high vorticity, high velocity and pressure fluctuations, which amplify locomotion costs and risk of disorientation. According to the experimental data reported by Müller *et al.* [25], the percentage of time fish spent in the turbine’s main wake area was minimal, as follows: HR (15%), HS (3%), MR (14%) and MS (8%). Previous studies have shown that flow past bluff bodies, such as cylinders, can create favourable microhabitats for fish. When turbulence exhibits appropriate intensity, orientation and spatial scale, fish may benefit from reduced muscle activity, thereby enhancing their swimming efficiency. Some species are known to exploit these flow conditions by station holding or engaging in Kármán gaiting within cylinder wakes [13,54–56]. However, the wake of the VAT, which is characterized by intense turbulence, swirling vortices and pronounced shear layers, appears less conducive to such energy-saving behaviours. So any potential energetic advantage from the reduced velocity is likely offset by the elevated TKE, underscoring the turbulence–velocity trade-off that influences fish behaviour in such environments.

The bow wake is another notable region located upstream of the VAT. In a flow past bluff bodies, such as bridge piers, the bow wake is a high-pressure zone where the flow velocity is reduced relative to the free stream [54]. Fish often exploit this low-momentum region for station holding. While fish were occasionally observed entering the turbine’s bow wake, this behaviour was relatively infrequent, probably owing to the elevated TKE in the upstream region. According to the experimental data [25], the percentage of time fish spent in the bow wake was limited to HR (2%), HS (1%), MR (2%) and MS (0%). Although localized low-velocity pockets within the bow wake may be attractive, the presence of high-intensity vortical structures tends to discourage occupancy. Overall, fish exhibit a clear preference for refuges with reduced turbulence, often located near channel peripheries, and tend to avoid high TKE wake flows of high turbulent shear stress that require greater energy for stabilization (figure 7a, gold; table 4, gold).

#### 3.2.3. Velocity adjustments and swimming effort

Figure 10 presents the distribution of fish swimming speeds across the defined flow subregions under both rotating and stationary turbine conditions. The results show that the mean swimming speed is highest in the far wake (subregion P11) and the bow wake (P5) under rotating turbine conditions compared with their stationary counterparts. Additionally, fish spent only a very small proportion of time in the near wake and bow wake subregions (P5 and P8), indicating that these regions are generally avoided. These observations suggest that fish adjust their swimming behaviour in response to turbine-induced turbulence and shear layers, tending to move through these regions more quickly rather than occupying them.

**Figure 10.**
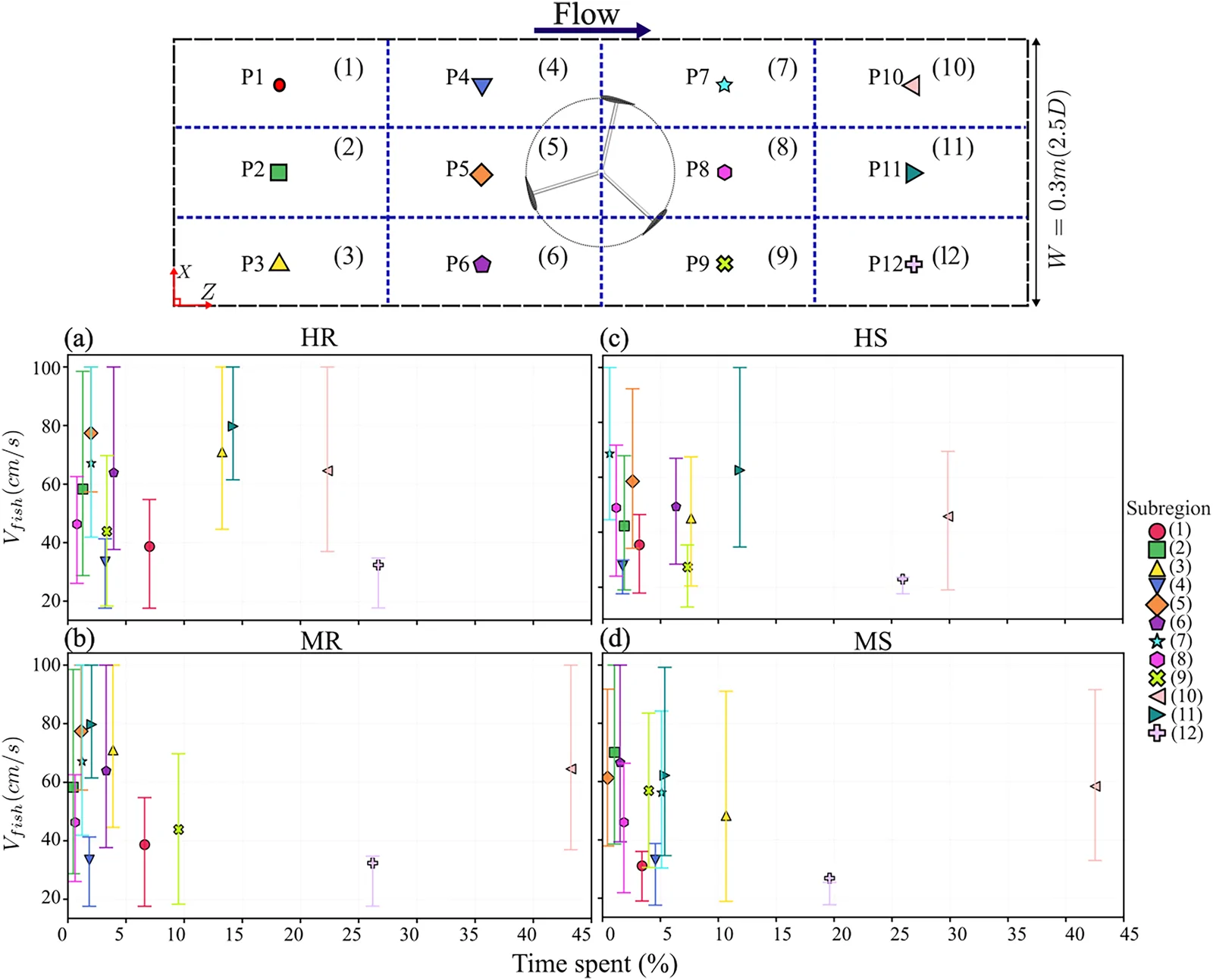
Schematic layout and subregion definitions (top) and scatter plots of mean absolute fish swimming velocity ($V_{\text{fish}}$) versus the percentage of time fish occupied each subregion for all experimental conditions: (a) HR, (b) MR, (c) HS and (d) MS. Each point corresponds to one of the 12 channel subregions (1–12). The horizontal axis indicates the fraction of total time fish spent in each subregion, and vertical error bars represent variability in swimming velocity in each subregion. Swim speeds are flow-adjusted, and velocities are capped at the critical burst swimming speed (CBSS) threshold of 100 cm s^–1^.

These patterns are consistent with the broader trends revealed by the LES-computed turbulence metrics. In particular, regions characterized by stronger TKE and vortical structures tend to correspond to areas where fish spend less time and exhibit higher swimming speeds, indicating more active swimming behaviour when swimming through energetically demanding flow environments.

Supporting evidence from the experimental dataset reported in [25] further indicates that across all test conditions, fish frequently maintained very low swimming speeds ($V_{\text{f}}\leq 0.03\;{\text{m s}}^{-1}$) or exhibited station holding behaviour, reflecting a clear energy-conservation strategy. The percentage of time fish maintained very low velocities or performed station holding was reported as HR (63%), HS (84%), MR (71%) and MS (71%). Under mild flow conditions, station-holding tendencies did not differ markedly between stationary and rotating turbine configurations. However, under high-flow conditions, fish spent more time station-holding when the turbine was stationary. By contrast, blade rotation generated elevated vorticity and turbulent shear stresses, which prompted more active swimming responses, including increased upstream swimming and higher mean swimming velocities (figure 10).

Importantly, fish energetic demand, closely tied to oxygen consumption, varies with the hydrodynamic characteristics of the ecosystem. This demand can be evaluated in terms of a fish’s critical swimming speed ($U_{\text{cr}}$), which represents the maximum speed a fish can maintain before switching from aerobic to anaerobic metabolism [57]. As reported in the literature, swimming speeds up to 20% of $U_{\text{cr}}$ are considered low effort, 50% medium efort and 80% high effort [58]. Herein, we assume a critical burst swimming speed of 100 cm s^–1^ for small trout. To interpret fish swimming performance, we classify velocity ranges as follows: speeds less than 20 cm s^–1^ are considered low, speeds between 20 and 50 cm s^–1^ are moderate, speeds between 50 and 80 cm s^–1^ are high and speeds between 80 and 100 cm s^–1^ are classified as near-burst. This categorization provides a reference framework for interpreting the swimming velocities in figure 10.

The observed fish behaviour in the wake flow clearly shows that fish attempt to maintain stability while navigating the turbulent flow. They do so by balancing the flow rate, turbine-induced turbulence and energetic constraints. Figure 10 underscores this relationship by depicting the zone-averaged fish velocity (measured) in multiple subregions [34]. The results illustrate how fish dynamically adjust their swimming speeds in response to local turbulence levels and velocity gradients. For example, under high flow conditions (figure 10a,c), swimming speeds are generally higher compared to those observed under mild flow conditions (figure 10b,d). This reflects the increased effort required to navigate stronger currents and more energetically turbulent environments. Additionally, in rotating cases (HR and MR; figure 10a,b, respectively), the turbine introduces elevated turbulence, particularly in the wake region, which contributes to both higher fish velocities and greater variability in swimming speeds. By contrast, stationary cases (HS and MS; figure 10c,d) exhibit more consistent velocities, as fish either maintain station more effectively or relocate to calmer regions. As a result, evasion manoeuvres in areas of high TKE are less frequent. In certain subregions near the turbine (e.g. 7–9), moderate average swimming speeds are accompanied by high percentages of upstream swimming. This pattern suggests that the fish tend to resist the current while also attempting to avoid direct exposure to the turbine wake.

To swim effectively, fish must generate a thrust force that counteracts hydrodynamic drag. This thrust requirement can serve as a useful metric for evaluating the physiological cost of swimming in the wake flow field induced by VAT. Based on the fish swimming speed derived from the fish experiments, the thrust force generated by the fish was estimated according to [24], where thrust force ($T_{f}$) and its unit for a swimming fish can be expressed as

(3.1) $$\begin{array}{r} T_{f}=\rho_{f}V_{f}^{2}L_{f}^{2}\;(\text{dyne = }g\;\mathrm{cm}\;s^{-2}), \end{array}$$

where $\rho_{f}$ is the fish’s density, $V_{f}$ is the swimming velocity and $L_{f}$ is the fish’s body length. We focus on a rainbow trout (*O. mykiss*) with an average body length of $L_{f}\approx 6\;\text{cm}$, used in the experiments [25]. The density of trout is considered $\rho_{f}\approx 1.08\;g\;{\mathrm{cm}}^{-3}$, which is slightly denser than water. These estimates enable calculation of thrust force across all subregions and further quantify the energetic demands associated with turbine-induced turbulence (table 5). As seen in table 5, even modest variations in fish swimming speed ($V_{f}$) result in substantial changes in $T_{f}$, owing to the quadratic dependence on velocity. In other words, swimming velocity significantly impacts the physiological cost of swimming. By comparing the test cases, one can assess the impact of bulk velocity and turbine rotation on fish swimming velocity and thrust requirements. Importantly, thrust force is directly tied to oxygen consumption and muscular exertion; subregions with high $T_{f}$ values correspond to areas of elevated energetic demand. Prolonged exposure to such zones may lead to muscle fatigue, prompting fish to seek calmer regions for station holding and energy conservation [24].

Autocorrelation analysis of the LES-computed streamwise velocity fluctuations revealed that the turbulence length to fish length ratio ($\Lambda /L_{\text{fish}}$) exceeded unity throughout much of the domain and ranged between 0.96 and 4.99 (table 6). Lower values ($\Lambda /L_{\text{fish}}\approx 1$–1.5) occurred primarily near refuge regions such as sidewalls, whereas the largest values ($\Lambda /L_{\text{fish}}>3$) were associated with the wake core and shear layer regions. Notably, in regions where fish were seen to exhibit abrupt trajectory changes and evasive manoeuvres (figure 9), the local $\Lambda /L_{\text{fish}}$ ratio exceeded approximately 1.5, suggesting that interaction with eddies larger than the fish body length contributed to the observed instability and rapid corrective swimming behaviour.

Previous experimental studies have shown that turbulent eddies with the characteristic length to fish length ratio exceeding approximately 0.5–0.75 can destabilize swimming posture and induce corrective manoeuvres [12,23,24]. In the present study, the threshold at which destabilization and sudden movements occur is higher than previously observed. The threshold will be dependent on many factors, including the eddy orientation in relation to the fish body, ‘background’ velocity magnitude, turbulent shear stress magnitude, fish species and life stage. Nevertheless our results suggest that turbulence length to fish length ratio could be used as a proxy to predict unfavourable (and favourable) flow regions in turbine-induced flows and a combination of metrics such as TKE, Reynolds shear stresses and velocity (table 4 and figure 7) could be better indicators for predicting the conditions that induce responses including adaptive swimming manoeuvres, rapid/evasive swimming responses and destabilization.

A key component of wake flow dynamics relevant to fish stability is the turbulence integral length scale, which characterizes the size of the largest energy-containing eddies in the flow. Turbulent flows consist of a hierarchy of eddies, ranging from the integral length scale—where most of the TKE resides—down to the Kolmogorov scale, at which viscous dissipation dominates. In this study, the integral length scale is estimated using velocity autocorrelation functions of the streamwise velocity fluctuations $\rho_{uu}$ [59,60]:

(3.2)ρuu(τ)=⟨u′(t)u′(t+τ)⟩⟨u′(t)u′(t)⟩,(3.3)T=∫0∞ρuu(τ)dτand(3.4)Λ=∫0∞ρuu(r)dr,

where T represents the temporal coherence of turbulence and Λ denotes the spatial integral length scale. Here, Λ is used as a proxy for the characteristic size of dominant energy-containing eddies interacting with the fish body.

Table 6 summarizes the computed integral length scales normalized by fish length, $\Lambda /L_{\text{fish}}$. Previous experimental studies have shown that turbulent eddies with characteristic length scales exceeding approximately 50–75% of fish body length can destabilize swimming and induce corrective manoeuvres [12,23,24]. The turbine-induced flow field and rainbow trout considered here ($L_{\text{fish}}=6$ cm), corresponds to values of $\Lambda /L_{\text{fish}}$ between 0.96 and 4.99 across all test cases, so exceeds unity throughout much of the domain. The refuge regions such as the sidewall regions exhibit lower values ($\Lambda /L_{\text{fish}}\approx 1$–1.5), whereas the largest values ($\Lambda /L_{\text{fish}}>3$) are associated with the wake core and shear layer regions where fish exhibited abrupt trajectory changes and evasive manoeuvres (figure 9). Thus, our results suggest that turbulence length to fish length ratio could be used as a proxy to estimate favourable regions over unfavourable regions in turbine-induced flows and a combination of turbulence metrics (e.g. magnitude of TKE, turbulence shear stresses, etc.; see figure 7 and table 4) could be better indicators of hydrodynamic conditions that induce destabilization, cause evasive or adaptive swimming manoeuvres and active swimming responses.

#### 3.2.4. Turbulence–velocity trade-off and evasion

The LES-computed flow field in the wake of the rotating turbines clearly indicates that, while the flow momentum in the wake is greatly decreased, the TKE increases significantly. Some fish appeared to exploit this low-momentum wake region for partial energy savings, albeit up to a certain threshold. This reduced velocity–elevated tubulence trade-off compels fish to spend more time within the wake of the rotating turbine compared to the stationary turbine case (figure 10). However, the wake occupancy time was less than that of the far downstream sidewalls. Spatial analysis of the occupancy time is reported in the experimental observation in [25]; the percentage of time fish entered and stayed in the rotor wake as: 0, 5, 10 and 10% for HR, HS, MR and MS cases, respectively. Furthermore the percentage of time fish occupied the high velocity region outside the rotor area attempting to travel upstream of the turbine was reported as: 35, 15, 30 and 20% for HR, HS, MR and MS cases, respectively. These metrics demonstrate two key aspects of fish–turbine interactions, that is, the frequency of fish remaining in the rotor wake and the proportion of time fish are positioned in the high-velocity region of the flow near the sidewalls, attempting to make their way to the upstream area. The low-velocity rotor wake region, and the high-velocity region outside the wake along which fish attempt to make upstream passage can be seen in blue and red colours of figure 2, respectively, and the highly turbulent region of the wake, marked by elevated absolute values of out-of-plane vorticity, can be seen in figure 4.

In the HR case, which is associated with an elevated turbulent intensity in rotor wake, fish never enter the rotor wake area (i.e. 0% occupancy). The high TKE in this region means that fish need to generate greater thrust and expend more energy while exposed to the risk of disorientation or injury. These deterrents appear to outweigh any potential benefits of occupying the rotor region. By contrast, the HS case with a 5% rotor wake occupancy suggests that the absence of rotation reduces turbulence to a level where fish occasionally explore the rotor zone. Under mild flow conditions (MR and MS) with mild turbulence levels in the rotor wake, the rotor wake entry increases to 10%, indicating that the reduced turbulence allows fish greater freedom to explore the region. On the other hand, the fish remained inside the high-velocity region of the flume for relatively longer times (35%), attempting to make passage to the upstream. This could be owing to strong wake turbulence and the high energetic cost of staying in the high-velocity region, which compels the fish to attempt upstream navigation. In the HS case, a relatively lower passage attempt rate inside the high-velocity region (15%) suggests fish can enter and explore the high-velocity region but are less frequently compelled to pass upstream. In mild conditions, passage attempts inside the high-velocity region reach 30% (MR) and 20% (MS), consistent with reduced turbulence and more favourable hydrodynamic conditions. However, turbine rotation in the MR case may introduce localized eddies or disturbances that prompt more rapid upstream passage rather than extended exploration.

These findings indicate that in high-flow rotating cases (HR), the strong shear layers, which separate the highly turbulent wake and high-velocity regions, act as a hydraulic barrier deterring fish from entering the rotor wake (i.e. 0% entry), yet prompting frequent passage attempts (35%) because of limited alternative routes. By contrast, under mild conditions, fish are more likely to enter the rotor wake region (10%) but may not attempt or complete passage owing to possibly cautious behaviour. From an ecological perspective, repeated upstream passage attempts through high-velocity regions could lead to energy depletion [55], increased fatigue and elevated predation risk. While high turbulence may reduce collision risk by discouraging entry, it concurrently impedes critical migratory movement. Conversely, low-turbulence environments (MS and HS) may allow safer exploration but do not guarantee prolonged occupation or successful upstream transit. These findings underscore the importance of striking a balance between turbine design and operation to minimize both physical hazards and behavioural disruptions to aquatic life.

#### 3.2.5. Fish passage and collision avoidance

In terms of evasion and collision avoidance, fish predominantly exhibited evasive manoeuvres in the rotating turbine scenarios under mild-flow conditions. Although the probability of collision increases when fish approach the rotor, TKE seems to act as a behavioural trigger, prompting fish to perform rapid evasive actions to maintain stability and avoid contact with the rotating blades. Under high-flow conditions, however, the elevated wake turbulence appears to act as a natural deterrent, reducing fish entry into the rotor zone and consequently lowering the risk of collision. As seen in the colour maps of the LES-computed vorticity field of figure 4, the absolute value of vertical vorticity is greatest near the blades and along the shear layer. Such elevated turbulence seems to be particularly useful in alarming the fish to avoid collision.

As observed in the experimental test, the percentage of time fish exhibited blade evasion manoeuvres was 20, 0, 35 and 0% for HR, HS, MR and MS cases, respectively [25]. In addition, the percentage of observed collisions with the turbine blade was: 5, 0, 25 and 0% for HR, HS, MR and MS cases, respectively. Evasive responses were most frequent in the MR case, where the mild flow conditions allowed for increased fish exploration, but the turbulence generated by the rotating blades triggered short-duration manoeuvres to avoid blade contact (figures 7 and 9). These behavioural adaptations are critical for mitigating mechanical injury, historically the primary focus of FIA. Furthermore, the prevalence of evasive manoeuvres near high I regions supports recent efforts to include turbulence-induced stressors in revised FIA frameworks. Notably, actual collisions were rare across all scenarios, particularly under high-flow conditions (HR and HS), where increased turbulence and flow velocity appeared to discourage fish from approaching the rotor altogether.

### 3.3. Implications for marine hydrokinetic design and environmental assessment

In narrow riverine channels that function as migratory corridors, the present framework suggests that assessments could examine whether a continuous, hydraulically accessible passage route remains outside the principal turbine wake. The observed associations indicate that energetic wake regions and shear layers may discourage passage by juvenile rainbow trout under controlled flume conditions. Turbine siting, lateral clearance, array layout and operating conditions could therefore be evaluated using velocity, TKE, Reynolds stresses, vorticity and $\Lambda /L_{\text{fish}}$ as candidate behaviourally relevant metrics. These implications should be treated as testable design considerations rather than universally applicable recommendations. Species- and site-specific validation is required, particularly before extending them to natural river systems or marine environments.

## 4. Conclusion

This study integrates high-resolution LES with experimental observations of juvenile rainbow trout around a VAT to investigate fish–turbine interactions in turbulent aquatic environments. The main focus is the integrated eco-hydrodynamic framework used to map experimentally observed fish trajectories onto turbulence-resolved LES fields and relate fish movement and spatial occupancy to local hydrodynamic conditions. Moving beyond conventional blade-strike assessments, we focus on the role of hydrodynamic and turbulence-induced stressors in shaping fish behaviour near hydrokinetic devices.

Using LES coupled with a CURVIB method, we resolved complex three-dimensional wake structures, including wake asymmetry, vortex shedding and spatially varying turbulence fields. These simulations enabled key turbulence metrics to be directly compared with experimentally observed fish trajectories. Fish consistently avoided regions of elevated TKE and shear and preferentially occupied low-turbulence regions near the channel boundaries. A key finding was the *velocity–turbulence trade-off*: fish occupied relatively higher-velocity regions when these regions had lower turbulence, indicating that hydrodynamic stability may be prioritized over minimizing swimming speed. Frequent evasive manoeuvres were also observed where the turbulence length scale was comparable to or larger than fish length, while thrust-force estimates indicated greater energetic demands within the rotor wake.

From an ecological perspective, elevated turbulence and shear may discourage rotor entry and reduce opportunities for blade contact, but these flow features may also form hydraulic barriers, increase swimming effort and interfere with upstream passage. Therefore, environmental assessments should consider passage continuity, avoidance behaviour, energetic demand and access to low-turbulence refuges alongside direct mechanical injury.

From an engineering perspective, these findings highlight the importance of turbine siting, operating conditions and environmental integration. Turbine installations should preserve continuous passage routes, while array design should consider the downstream development and interaction of TKE, Reynolds stresses, vorticity and turbulence length scales. Overall, incorporating behaviourally relevant turbulence metrics into assessment frameworks can support the design and operation of hydrokinetic systems that reduce mechanical injury risks and hydrodynamic stressors while preserving aquatic ecosystems.

## Supplementary Material



## Figures and Tables

**Figure 11.**
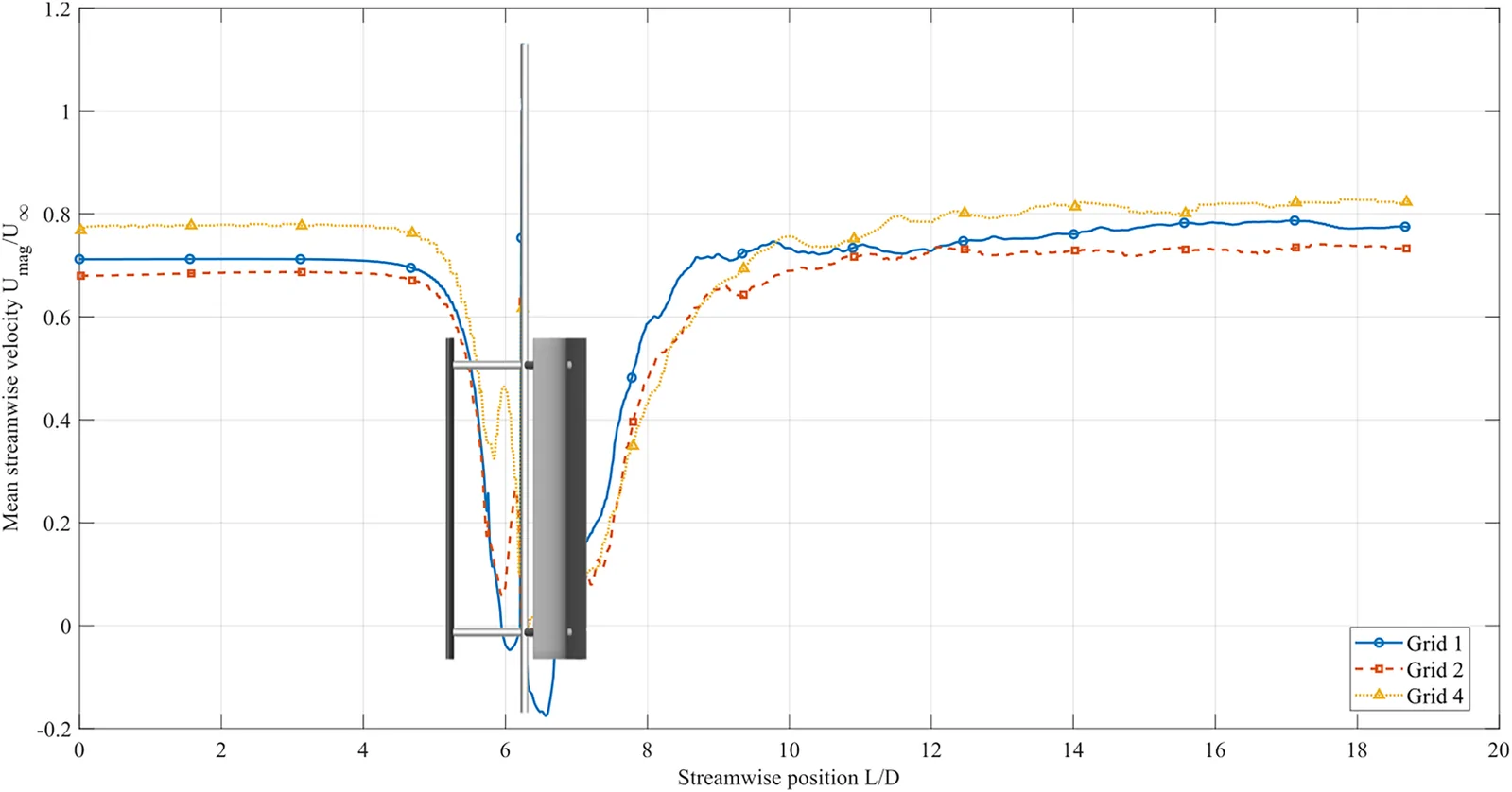
Variations of normalized mean streamwise velocity in the streamwise direction. The solid, dashed and dash-dotted curves with distinct markers represent results obtained from grid systems 1, 2 and 4, respectively. The streamwise coordinate is normalized by the rotor diameter (D). The rotor region is specified by inserting a schematic of the turbine onto the figure.

**Table 7.** Computational grid specifications used in the grid-sensitivity analysis. Grid dimensions are reported in the spanwise, vertical and streamwise directions. The characteristic near-turbine spacing is normalized by turbine diameter (D). The reported $y^{+}$ values correspond to representative values at the first off-wall point near the turbine blades. Grid A was used for the production simulations.

variable	grid A	grid B	grid C
node count	$22\times 10^{6}$	$5.7\times 10^{6}$	$1.2\times 10^{6}$
$N_{x},N_{y},N_{z}$	217×121×837	109×125×421	57×65×321
$\Delta_{x},\Delta_{y},\Delta_{z}$	0.0083D	0.0166D	0.0333D
$\Delta t^{*}$	0.0005	0.0005	0.0005
$y^{+}$	12	24	48

**Figure 12.**
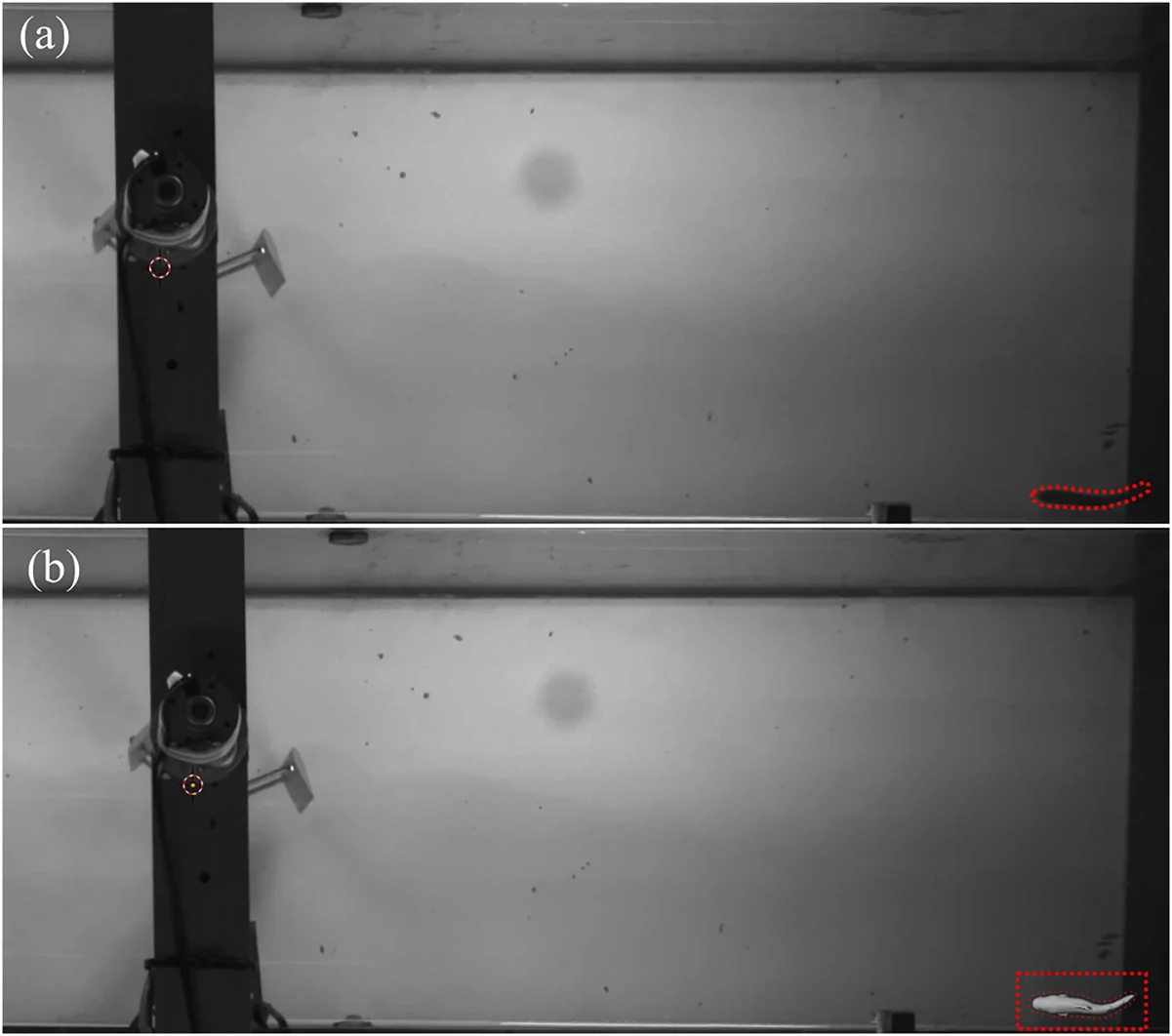
Timeframes illustrating the digitization process of live fish from experimental observations. (a) The live rainbow trout, which is outlined in red, used in the experiments. (b) The digitization of the fish’s location and body posture into an object, which is then saved as a snapshot. These snapshots are later superimposed onto the LES results to analyse fish–turbine flow interactions.

## Data Availability

The computational model’s code is openly available at [61]. The hydrodynamic simulation results can be accessed at [62], and fish analysis data, including trajectories, velocities and turbulence mapping, derived from experiments and numerical simulations, are available at [63]. Supplementary material is available online [64].

## Footnote

^1^

https://blender.org.

## Appendix A. Grid sensitivity analysis

A grid independence study was conducted to evaluate the effect of mesh resolution on the predicted mean flow characteristics and to determine an optimal grid resolution that provides sufficient accuracy and turbulence resolution while maintaining numerical efficiency. Three computational structured grids—A, B and C, with characteristic cell sizes of 1 mm (Grid 1), 2 mm (Grid 2) and 4 mm (Grid 4) near the turbine region—were employed, as summarized in table 7. The comparison of the streamwise evolution of the normalized mean velocity along the turbine centreline, $U_{\text{avg}}$, for the MR test case is presented in figure 11 (table 3).

All grids exhibit nearly identical upstream velocity profiles, whereas the largest discrepancies appear in the near-wake region, where the flow deceleration and subsequent recovery are most pronounced. As the grid is refined from 4 to 1 mm, the minimum velocity deficit deepens slightly, and the wake recovery becomes smoother and more physically consistent. At the downstream, all profiles converge, indicating that the flow field becomes nearly grid-independent. Based on these observations, Grid 1 (1 mm) was selected for the final simulations, as it provided sufficient resolution to accurately capture the wake dynamics while ensuring numerical stability and grid-independent results.

## Appendix B. Fish tracking and supplementary video construction

Fish behaviour was recorded using a fixed Baumer camera mounted above the narrow flume. The camera acquired monochrome images at approximately 80 fps, with an image resolution of 600×2352 pixels [25]. The recorded test section extended 10D=1.20 m in the streamwise direction and 0.30 m in the spanwise direction. Because the single-camera arrangement introduced some image skew and distortion, the extracted coordinates were scaled to the known dimensions of the experimental test section before analysis. The top-view arrangement provided planar fish positions in the streamwise and spanwise directions; swimming depth was not resolved [25].

The resulting experimental video, an example of which is available in the supplementary material of [25], provided the observational input for the present eco-hydrodynamic framework. The video was first processed at 4 Hz using the open-source Animal Tracker plugin in ImageJ to extract the planar fish position and body outline at each analysed frame. The experimental video and extracted trajectory were then imported into Blender. As illustrated in figure 12, the live fish was identified in each experimental frame (figure 12a), and a virtual fish was placed at the corresponding measured position and oriented to reproduce the observed body posture (figure 12b). This procedure was repeated for the selected frames to create the sequence of snapshots used in the supplementary animation.

Tracking records were visually inspected against the original image sequence. Frames affected by shadows, fish proximity to the sidewalls or particles moving through the field of view were manually corrected when the fish position could be identified reliably.

For video 1 in the supplementary material, a representative fish trajectory from the mild-flow rotating case was imported into Blender. The experimental trajectory was resampled at intervals of 0.9917 s, and the fish outline was placed at the corresponding position within the numerical representation of the flume. The underlying experimental record represents 600 s of physical time.

The turbulence field shown in supplementary material, video 1, consists of instantaneous LES-computed Q-criterion isosurfaces at Q=50, coloured by instantaneous velocity magnitude. The field is therefore instantaneous rather than time averaged. The fish trajectory and LES flow field were generated independently and were not temporally synchronized. However, the LES reproduced the experimental flume geometry, water depth, bulk velocity, turbine operating state, and tip-speed ratio and was sampled after statistically converged flow conditions were established. Accordingly, the instantaneous LES fields represent characteristic wake realizations under the corresponding experimental condition rather than the exact turbulent structures encountered by the displayed fish.
