# Supplementary material for: On the interaction of fish and marine hydrokinetic turbines: insights gained through experimental and computational observations
Source: R Soc Open Sci. 2026 Nov 11;13(11):262007. doi: 10.1098/rsos.262007 (PMC13750931; doi:10.1098/rsos.262007)
Supplement: Supplementary file 1 [file rsos.262007.zip › rsos.262007/rsos262007_si_002.docx]

Supplementary materials

<https://www.youtube.com/embed/j78yZIIqczg?feature=oembed>

Video 1- Video animation of fish trajectory superimposed by the LES-computed iso-surfaces of the Q-criterion, colored by normalized velocity magnitude ($u_{mag}/U_{\infty}$). The video is constructed from snapshots of the flow for 600 s of physical time.
